# A near infrared light emitting electrochemical cell with a 2.3 V turn-on voltage

**DOI:** 10.1038/s41598-018-36420-1

**Published:** 2019-01-18

**Authors:** Babak Nemati Bideh, Hashem Shahroosvand, Ahmad Sousaraei, Juan Cabanillas-Gonzalez

**Affiliations:** 1 Group for Molecular Engineering of Advanced Functional Materials (GMA),Chemistry Department, University of Zanjan, Zanjan, Iran; 2 Madrid Institute for Advanced Studies in Nanoscience, IMDEA Nanociencia, Calle Faraday 9, Ciudad Universitaria de Cantoblanco, 28049 Madrid, Spain

## Abstract

We report on an organic electroluminescent device with simplified geometry and emission in the red to near infrared (NIR) spectral region which, has the lowest turn-on voltage value, 2.3 V, among light emitting electrochemical cells (LEECs). We have synthesized and characterized three novel ruthenium π-extended phenanthroimidazoles which differ on their N^N ligands. The use of dimethyl electron donating groups along with the π-extended phenanthroimidazole moiety promotes ambipolar transport thereby avoiding the use of additional charge transport layers. Furthermore, a facile cathode deposition method based on transfer of a molten alloy (Ga:In) on top of the active layer is deployed, thus avoiding high vacuum thermal deposition which adds versatile assets to our approach. We combine ambipolar charge transport organic complex design and a simple ambient cathode deposition to achieve a potentially cost effective red to NIR emitting device with outstanding performance, opening new avenues towards the development of simplified light emitting sources through device optimization.

## Introduction

In recent years, the search for new functional materials with emissive properties in the NIR region attracted intensive attention. However, NIR electroluminescence (EL) is generally limited to rare reports, a consequence of the difficulties to overcome the intrinsically lower radiative decay rate of excited states as the energy band gap shrinks. This is reflected in low NIR external quantum efficiency (EQE) values in the 0.1% range^1–5^. In order to obtain efficient NIR emission and overcome the energy gap limitation, it is necessary to utilize specific ligands capable of extending the π-electron delocalization of the aromatic chromophore and bearing the substitution of nitrogen by less electronegative carbon atoms in benchmarked cyclometalated emitters^6–10^. Ruthenium polypyridyl complexes have attracted substantial interest for NIR EL because of novel and important applications such as bio-imaging^6,7^, telecommunications^11,12^, and wound healing^11,13^. Reports on NIR EL based on other metal complexes are less numerous, with the exception of few examples such as Iridium cyclometalated complexes^14,15^. The use of complexes based on benchmark lanthanide diketons such as Nd, Er, Pr, Yb, Ho, and Tm is jeopardized by their low emission quantum efficiency (ϕ_PL_∼10^−4^–10^−6^) due to coupling of the 4f excited states in the lanthanide ions with ligand vibrational modes and other re-absorption processes^16^. Moreover, the well-known environmental impact associated to lanthanide mining limits their extensive applications to this field. In response to the intense appeal for efficient and inexpensive NIR light-emitting devices, LEECs appear as promising candidates thanks to their simplicity, not requiring the use of electron and transport layers to balance charge transport in contrast to OLEDs^17,18^, and their notable EL performance^19^. Moreover, LEECs exhibit the interesting feature of emission tunability through replacement of the ionic transition metal in the complex. In addition, NIR EL can be further promoted by appropriate ligand design according to five strategies: (i) the design of ligands with large steric hindrance to avoid energy relaxation via resonant vibrations^20,21^. (ii) The incorporation of electron donating groups to the ancillary ligand to decrease the band gap through HOMO destabilization or LUMO stabilization^22,23^. (iii) The triplet energy level matching of the ancillary ligand with the LUMO of the metal center to create efficient electron transfer^24–27^. (iv) The extension of the ancillary ligand π-conjugation to reduce the band gap and cause emission red shift^28,29^. (v) The addition of electronic conductive polymers such as poly(3,4-ethylenedioxythiophene) polystyrene sulfonate (PEDOT:PSS) to enhance the electron donor ability, leading to the increasing of electroluminescence characteristics^30^. The operation of LEECs relies on the movement of cations and anions towards the electrodes causing promotion of the charge injection properties. This generally leads to sufficient applied voltage to overcome the low ionic conductivity of the solid film^31^. The value of the turn on voltage is a key operational parameter in electroluminescent devices. In LEECs this parameter shows the rate of mobility of ions between cathode and anode when the voltage is applied across the device^32^. Although the optimization of the operational parameters in LEECs has attracted a great interest in recent years, there are not many reports targeting to reduce the turn on voltage in near infra-red LEECs, in contrast with abundant attempts on OLEDs (please see a short Review on the turn on voltage values in Ir/Pt/Ru polypyridyl complexes in ESI. Table S1). In this article, we describe the design of ruthenium complexes based on π- extended phenanthroimidazole.

**Figure 1:**
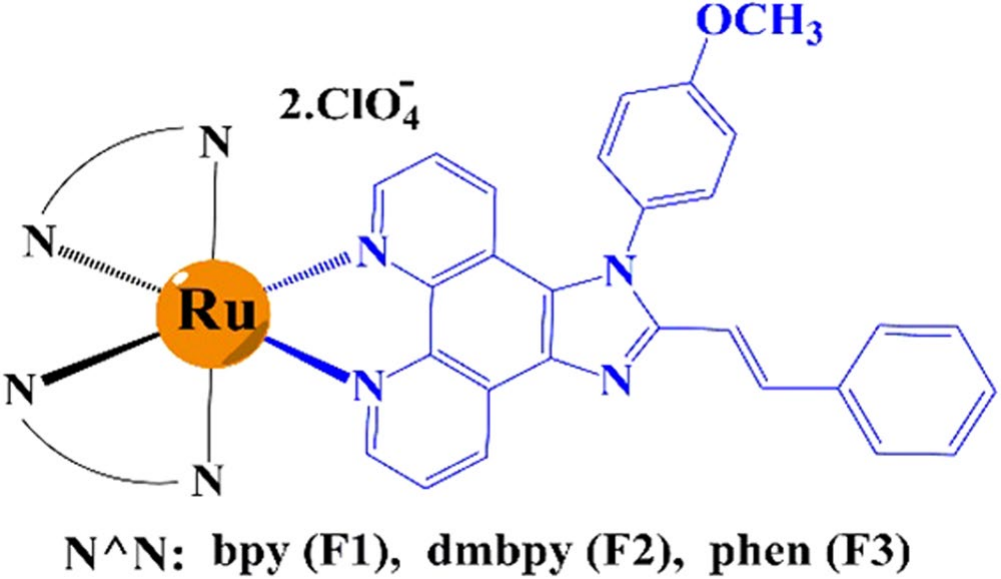
Chemical structures of the novel ruthenium (II) complexes with bpy, dmbpy and phen as ancillary ligand (black part). Blue part shows the structure of phenanthroimidazole moiety which repeats in all three complexes.

**Figure 2:**
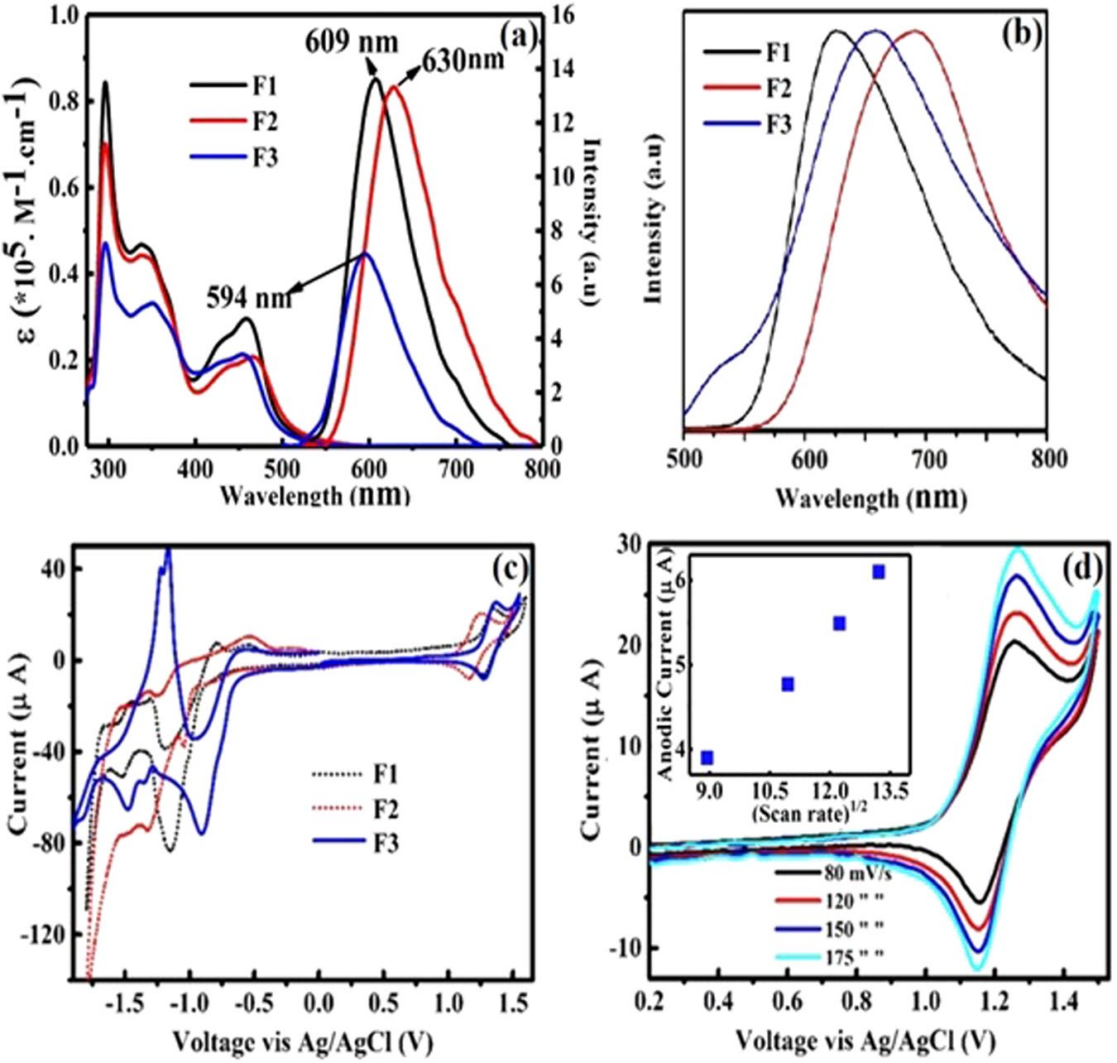
(**a**) UV-Vis spectra and PL spectra of complexes in acetonitrile solution. (**b**) Solid state PL of complexes supported on ITO glass substrates, (**c**) CV of F1-F3 at scan rate of 0.08 V/s, (**d**) CV of the of F2 at various scan rates of 0.08, 0.12, 0.15, 0.175, V s^−1^. Inset: variation of I_p,a_ (anodic current peak).

These complexes exhibit Ru(III/II) based quasi-reversible characteristics and favourable optoelectronic properties for solid-state lighting. LEECs based on these complexes show efficient NIR EL with ultra-low turn on voltages which are, to the best of our knowledge, comparable to the lowest reported in light emitting devices (see Table S1 in ESI for turn on voltage values of metal transition complexes). The structure of the π-extended phenanthroimidazole ligand and its complexes containing different ancillary ligands including 2,2′-bipyridyl (bpy), 1,10-phenanthroline (phen) and 4, 4′-dimethyl 2,2′, bipyridyl (dmbpy), (namely F1-F3 respectively), are depicted in Fig. 1 and characterized in detail (ESI. S1–S9). The UV-vis absorption and photoluminescence (PL) spectra of the investigated complexes in solution are shown in Fig. 2a. The UV-vis absorption spectra of F1–F3 is typical for ruthenium polypyridyl complexes, with absorption bands in the UV spectral range (peaks at 300 and 350 nm) and in the visible (peak at 450 nm) which are attributed to intra-ligand charge transfer (ILCT) and metal-to-ligand charge transfer (MLCT) states respectively^33,34^. The broadening and red-shift of the MLCT band can be assigned to spin-orbit coupling^35^. The PL spectra of complexes (Fig. 2a,b) both in solution and film clearly show the influence of ancillary ligands which lead to λ_max_ emission peak wavelengths in F1, F2 and F3 of 609, 630 and 594 nm in solution and 628, 700 and 631 nm in solid film respectively (Table 1). The solid state PL shift is caused by the change in dielectric constant from solution to film^36–39^ as well as some degree of aggregation. Indeed, the PL spectra of crystalline powders (ESI. Fig. S10) exhibit an even further PL red shift with peaks at 650, 678 and 668 nm for F1–F3 respectively. A weak shoulder at 510 (526 nm) appears in the PL spectrum of F3 in solution (film).

**Table 1:** Photophysical and electrochemical data of F1–F3.

Comp.	Absorbance^a^ λ_max_(logε)	Emission λ_max_		Ru(II/III) Oxi.	E_0-0_^f^	E_HOMO_^g^	E_LUMO_^h^	E_gap_^i^
	Ligand Transitions MLCT	Solution^b^ (φ^c^)	Film^d^	E_1/2_ (ΔE) (V)^e^				
F1	296 (4.97), 336 (4.36), 458 (4.28)	609 (0.116)	626	1.30 (0.070)	2.30	−5.67	−3.37	2.30
F2	297 (4.93), 338 (4.33), 467(4.26)	630 (0.099)	689	1.20 (0.081)	2.24	−5.57	−3.33	2.24
F3	297 (4.89), 351 (4.21), 454 (4.23)	594 (0.088)	631	1.31 (0.080)	2.36	−5.68	−3.32	2.36
[Ru(bpy)_3_]^2+^	245 (4.40), 290 (4.91), 451 (4.17)	607 (0.095)	648	1.29 (0.079)	2.32	−5.66	−3.34	2.32

^a,b^In CH3CN [nm]. ^c^PLQYs were determined by comparison with [Ru(bpy)3]^2+^ (**φ** = **0**.**095**). ^d^ITO thin film of complexes (thickness of 90 nm). ^e^The E1/2 was obtained in CH3CN with 0.1 M TBAClO4 vs. Ag/AgCl. ^f^From the intersection of absorption and emission spectra. ^g^EHOMO = −(E1/2(vs. Fc/Fc+) +4.8). ^h^ELUMO = EHOMO + E0-0. ^i^Egap = EHOMO-ELUMO.

The trend of λ_max_ of all absorption and PL spectra in solution and film indicates the electron donating ability of the F2 dimethyl moieties causing an emission red shift compared to the other two ruthenium π-extended phenanthroimidazole complexes, an experimental confirmation of the band gap reduction (Table 1). Furthermore, DFT calculation show the reduction of band gap of F2 compared with other complexes (Fig. S11 in ESI, Table S2). A crucial factor for LEECs is the reversibility of the Ru(II)-Ru(III) redox reaction in ruthenium complexes^40^. Cyclic voltammograms (CV) of the three complexes (F1–F3) show a completely reversible behaviour in the positive voltage region assigned to Ru(II) to Ru(III) Ox/Red (Fig. 2c). The negative voltage region in the CV is attributed to the redox behaviour of the ligand which is characteristic of the ruthenium polypyridyl complexes^41^. The potential values of the Ru(II)-Ru(III) redox couples (${{\rm{E}}}_{{\rm{OX}}}^{1/2}$) are 1.30, 1.20 and 1.31 V for F1, F2 and F3 respectively, the lower F2 value being caused by the dimethyl electron donating group in the F2 ancillary ligand. Fig. 2d shows the CV of F2 at different voltage scan rate (ν).

The linear dependence of the anodic current with ν^1/2^ (inset in Fig. 2d) confirms that the kinetics of the process is controlled by mass transport. The electron transfer rate is, at all potentials, greater than the mass transport rate and the peak potential is independent of the applied voltage scan rate. The PL quantum yield (PLQY) values of F1-F3 in solution were 0.116, 0.099 and 0.088, respectively, with F1 and F2 having higher PLQE than [Ru(bpy)_3_]^2+^ (0.095).

**Figure 3:**
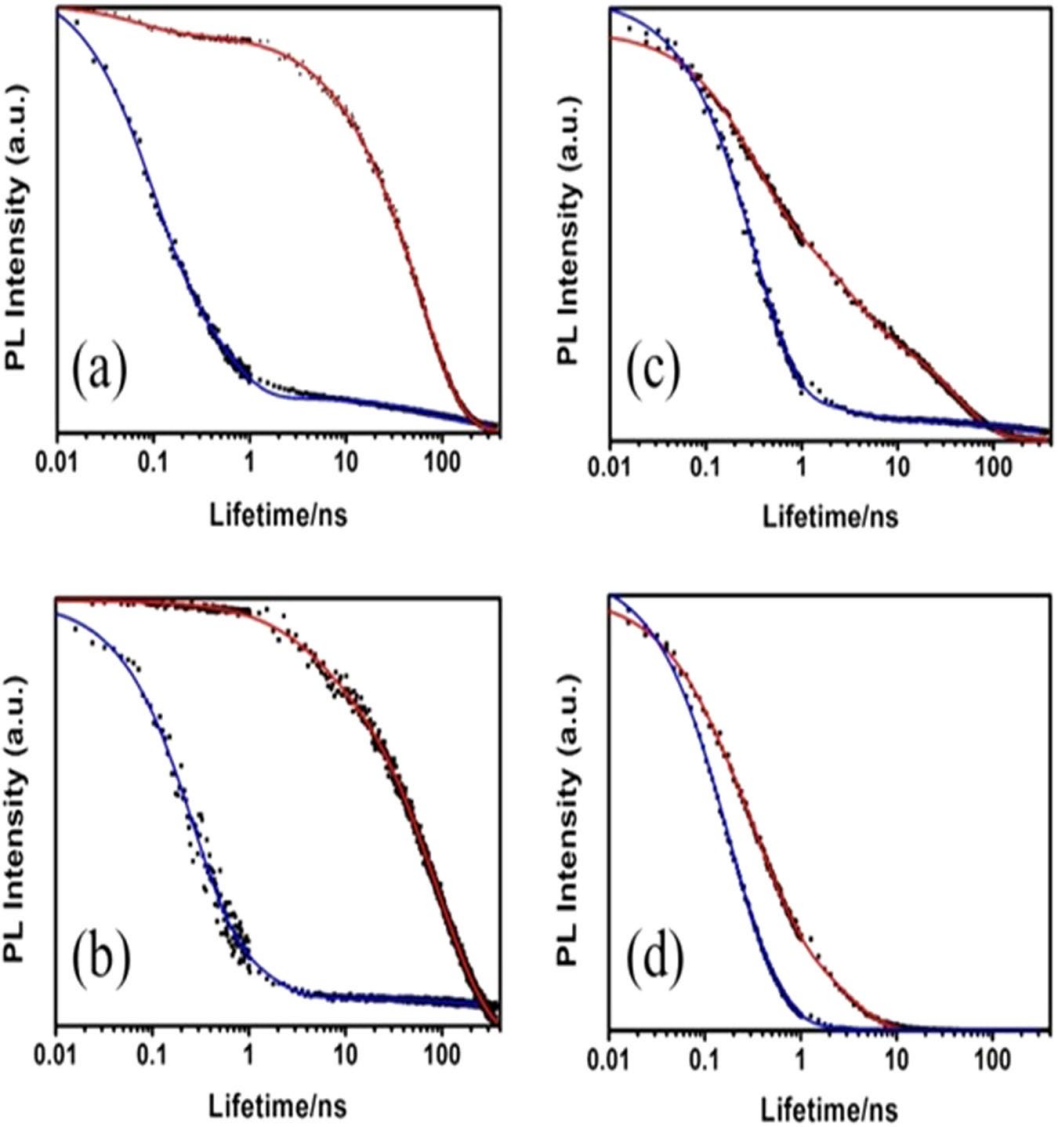
Time resolved photoluminescence measurements of (**a**) F1, (**b**) F2 and (**c**) F3 in ACN solution (dots, red lines) and film (dots, blue lines) detecting at 625 nm (solution) and 660 nm (film). (**d**) PL decay of F3 detecting at 510 nm (solution) and 526 nm (film). Straight lines stand for fits obtained with a three-exponential decay model.

Figure 3 displays the PL decay curves in solution and film of F1-F3. All samples were fitted with reasonable agreement to a three-decay exponential law with lifetimes spanning from the 0.1 ns to the 100 ns range (ESI. Table S3), such latter long component being characteristic of MLCT^3^ phosphorescence^42^.

Focusing on the PL lifetimes measured at the respective emission peaks (625, 690 and 660 nm for F1, F2 and F3 respectively) the average lifetimes of F1 and F2 (5.5 and 5.7 ns respectively) are longer than the corresponding of F3, (5.0 ns with predominant 0.3 ns lifetime). For more details on the individual lifetime components and their statistical weights please refer to Table S4 in ESI. Concomitantly, the short wavelength shoulders seen in the PL spectra of F3 in solution and film have sub-ns average lifetimes associated (0.5 and 0.9 ns respectively). Based on this short-lived nature, we speculate with the coexistence of two radiative decay mechanisms in F3 associated to emission from triplet MLCT^3^ states^43–46^ and residual ligand emission^47^. Comparing the PL lifetime values in solution and film, the latter are much shorter probably due to the effects of diffusion-assisted recombination of MLCT^3^ in solid state^48^.

**Figure 4:**
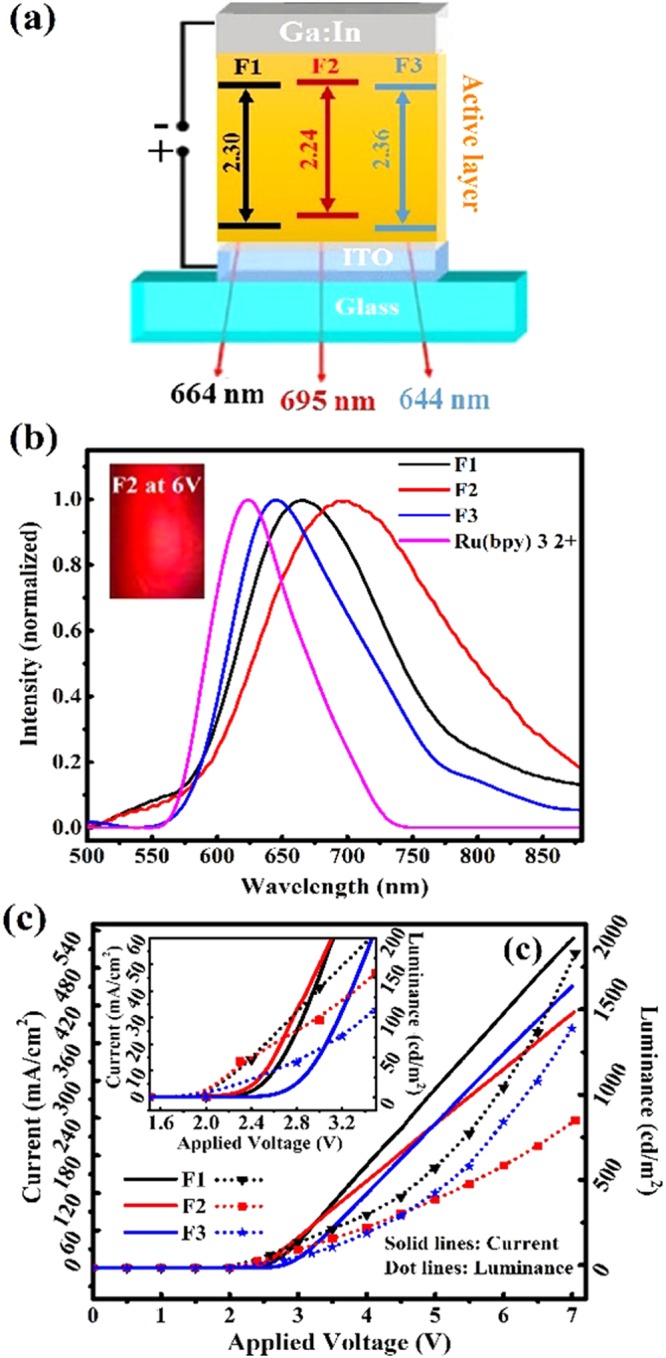
(**a**) Schematics of the LEEC structure with the band gap values of F1-F3 obtained from electrochemical data (**b**) EL spectra of ITO/F1-3/Ga:In devices. Inset: The deep red colour of F2 emitted by a pixel at 6 V (**c**) Current density and luminance over applied voltage for F1-F3 complexes. The same curves with expanded scale axis are shown in the inset.

The average lifetimes of F1, F2 and F3 in film decreased dramatically respect to solution (namely by a factor of 10, 16 and 2.5 respectively). Among the possible reasons for faster PL decay in film could be triplet quenching by traps (oxygen, film morphology) or triplet-triplet annihilation^49^. Next, we developed LEEC devices with a indium tin oxide (ITO)/complex/Ga:In configuration, (Fig. 4a). The use of a Ga:In molten alloy as cathode avoids using high vacuum thermal deposition which has a number of disadvantages such as mechanical stress due to the mismatch of the metal and organic layer thermal expansion coefficients and more importantly, the elevated temperatures reached during the thermal deposition process which puts restrictions on the coated substrates^50^.

**Figure 5:**
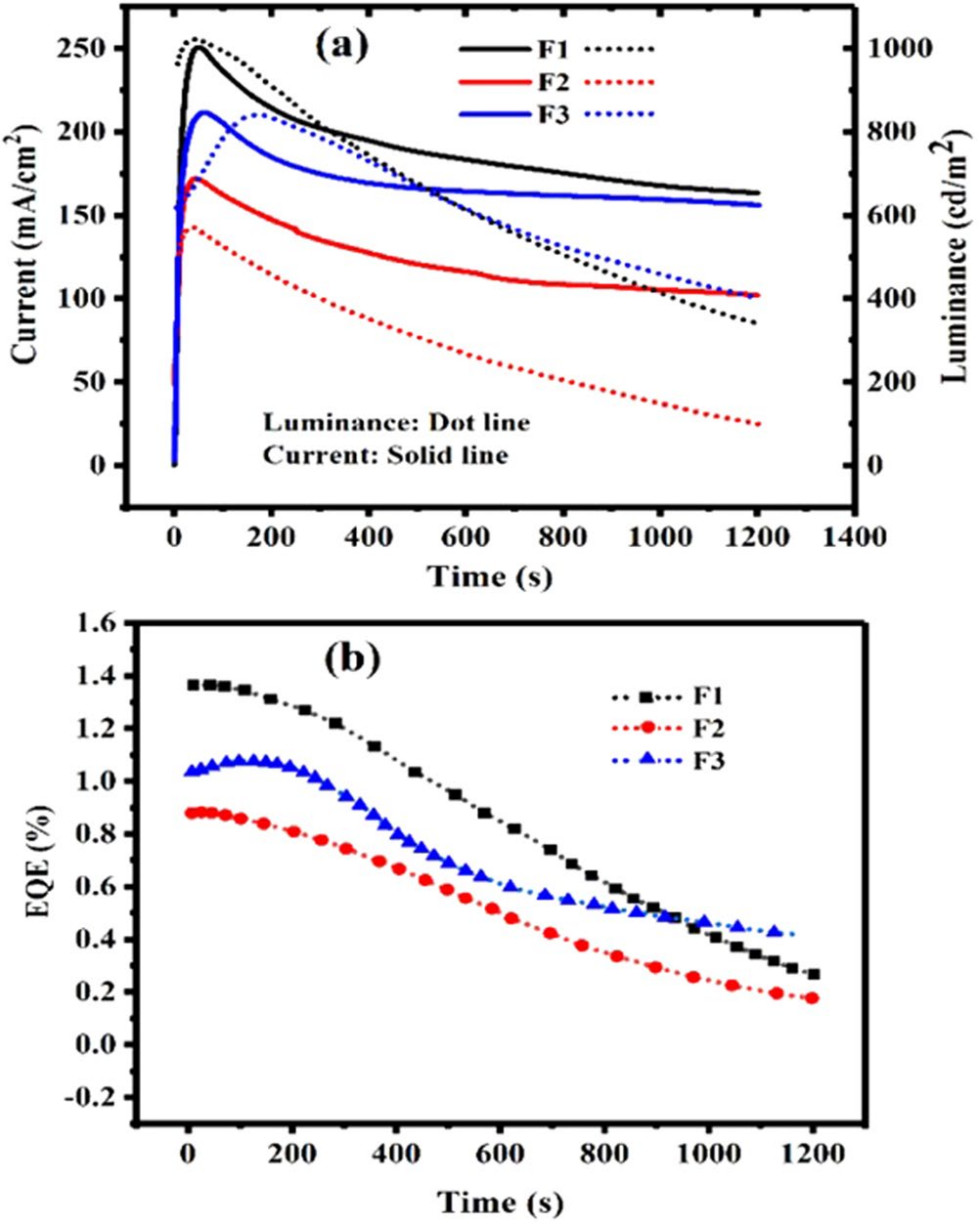
(**a**) Luminance and current over time at constant voltage of 6 V, (**b**) External quantum efficiency (EQE) over time at 6 V.

Figure 4b shows the EL spectra of LEECs based on F1-F3 in comparison with [Ru(bpy)_3_]^2+^. The peak and width of the EL spectra of F1-F3 follows the same tendency as the PL spectra, wherein F2 shows the broadest and longest wavelength peaked EL spectrum (720 nm), confirming the bathochromic effect of the dimethyl electron donating group. We remark that the EL of F1-F3 are all red shifted respect to [Ru(bpy)_3_]^2+^, due to the effect of the π-extended moiety on phenanthroimidazole. The I–V and Luminance-V results indicate that F1 exhibits larger current densities (540 mA/cm^2^) and luminance values (2100 cd/m^2^) measured at 7 V than F2 and F3. Fig. 5a shows current and luminance versus operation time, confirming the stability of all three LEECs. The higher device stability in F3 can be attributed to bulky effects of ancillary ligand^39,51^. We investigated the thermal stability of newly synthesized complexes by differential scanning calorimetry (DSC) analysis and found that they show high thermal stability with Tg = 234.9 °C and Tc = 275 °C. Moreover, the melting point of complexes is higher than 350 °C (ESI. S12). Outstandingly, the turn on voltage values found for F1–F3 amount only to 2.4, 2.3 and 2.8 V. These low values arise from the enriched electron density of the F1-F3 complexes, enabling the use of single layer architectures and significant device simplification. The 2.3 V turn on voltage achieved with F2 is only matched by Bard *et al*. with a ITO/[Ru(bpy)_3_]^2+^/Ga:In LEEC with red EL (660 nm)^37^, (see ESI and Table S1 for more details). The turn-on times obtained for F1, F2 and F3 were 36, 23 and 168 s respectively. In addition, we obtained device lifetimes of 793, 528 and 1125 s for F1, F2 and F3 respectively, (Table 2, Fig. 5a). Furthermore, the EQE evaluation along time suggested a better efficiency stability of F3 compared to the other two complexes (Fig. 5b).

**Table 2:** Electroluminescence data of LEEC device of ITO/(F1–F3)/Ga:In.

comp.	λ_max_(nm)	CIE^a^	FWHM(nm)	J^b^	${{\bf{L}}}_{{\bf{\max }}}^{{\bf{c}}}$	${{\bf{V}}}_{{\bf{on}}}^{{\bf{d}}}$	${{\bf{t}}}_{{\bf{on}}}^{{\bf{e}}}$	${{\bf{t}}}_{1{\boldsymbol{/}}2}^{{\bf{f}}}$	E^g^	EQE (%)^h^
F1	664	[0.730, 0.269]	128	3910	1066	2.4	36	793	0.27	1.40
F2	695	[0.734, 0.265]	170	3025	589	2.3	23	528	0.19	0.93
F3	644	[0.722, 0.277]	115	3405	878	2.8	168	1125	0.26	1.15
[Ru(bpy)_3_]^+2^	632	[0.710, 0.289]	80	4030	2500	2.3	—	—	0.65	—

^a^CIE(x,y): Commission Internationale de L’Eclairage, ^b^Current density(A m^−2^) at 6 V, ^c^Luminance(cd m^−2^) at 6 V. ^d^Turn-on voltage(V). ^e^Turn on time (second). ^f^Lifetime (second). ^g^Efficacy (cd A^−1^) at 6 V. ^h^External quantum efficiency at 6 V.

In conclusion, our results suggest that efficient NIR EL at very low turn on voltage (2.3 V) can be achieved with a purposeful design of ruthenium complexes based on a π-conjugated phenanthroimidazole ligand along with the substitution of electron donating groups on the ancillary ligand. Low turn on voltage values are demonstrated in single layer devices sandwiched between ITO and Ga:In, thus without requirements of additional charge transport and injection layers in contrast with the ever more complex OLED emitting architectures. The combination of electron acceptors with large π-delocalization groups enables then to extend the EL to the NIR region.

## Electronic supplementary material

ESI

## References

[1] Bolink HJ, Cappelli L, Coronado E, Gaviña P (2005). Observation of electroluminescence at room temperature from a ruthenium (II) bis-terpyridine complex and its use for preparing light-emitting electrochemical cells. Inorganic chemistry.

[2] Bolink HJ (2009). Deep-Red-Emitting Electrochemical Cells Based on Heteroleptic Bis-chelated Ruthenium (II) Complexes. Inorganic chemistry.

[3] Hosseini AR (2005). Addition of a phosphorescent dopant in electroluminescent devices from ionic transition metal complexes. Chemistry of materials.

[4] Wang S, Li X, Xun S, Wan X, Wang ZY (2006). Near-infrared electrochromic and electroluminescent polymers containing pendant ruthenium complex groups. Macromolecules.

[5] Xun S, Zhang J, Li X, Ma D, Wang ZY (2008). Synthesis and near-infrared luminescent properties of some ruthenium complexes. Synthetic Metals.

[6] Slooff L (2001). Near-infrared electroluminescence of polymer light-emitting diodes doped with a lissamine-sensitized Nd 3+ complex. Applied physics letters.

[7] Tessler N, Medvedev V, Kazes M, Kan S, Banin U (2002). Efficient near-infrared polymer nanocrystal light-emitting diodes. Science.

[8] Pal AK, Hanan GS (2014). Design, synthesis and excited-state properties of mononuclear Ru (II) complexes of tridentate heterocyclic ligands. Chemical Society Reviews.

[9] Pal AK, Serroni S, Zaccheroni N, Campagna S, Hanan GS (2014). Near infra-red emitting Ru (II) complexes of tridentate ligands: electrochemical and photophysical consequences of a strong donor ligand with large bite angles. Chemical Science.

[10] Hwang F-M (2005). Iridium (III) complexes with orthometalated quinoxaline ligands: subtle tuning of emission to the saturated red color. Inorganic chemistry.

[11] Bünzli J-CG, Eliseeva SV (2010). Lanthanide NIR luminescence for telecommunications, bioanalyses and solar energy conversion. Journal of Rare Earths.

[12] Curry R, Gillin W (1999). 1.54μm electroluminescence from erbium (III) tris (8-hydroxyquinoline)(ErQ)-based organic light-emitting diodes. Applied physics letters.

[13] Rausch AF, Thompson ME, Yersin H (2009). Blue light emitting Ir (III) compounds for OLEDs-new insights into ancillary ligand effects on the emitting triplet state. The Journal of Physical Chemistry A.

[14] Ertl CD (2017). Highly stable red-light-emitting electrochemical cells. Journal of the American Chemical Society.

[15] Pal AK (2017). Simple design to achieve red-to-near-infrared emissive cationic Ir (iii) emitters and their use in light emitting electrochemical cells. RSC Advances.

[16] Yersin, H. *Highly efficient OLEDs with phosphorescent materials*. (John Wiley & Sons, 2008).

[17] Xu Y (2018). Efficient Optical Gain from Near‐Infrared Polymer Lasers Based on Poly [N‐9′‐heptadecanyl‐2,7‐carbazole‐alt‐5, 5‐(4′,7′‐di‐2‐thienyl‐2′,1′,3′‐benzothiadiazole)]. Advanced Optical Materials.

[18] Costa RD (2012). Luminescent ionic transition‐metal complexes for light‐emitting electrochemical cells. Angewandte Chemie International Edition.

[19] Juris A (1988). Ru (II) polypyridine complexes: photophysics, photochemistry, eletrochemistry, and chemiluminescence. Coordination Chemistry Reviews.

[20] Imbert D, Cantuel M, Bünzli J-CG, Bernardinelli G, Piguet C (2003). Extending lifetimes of lanthanide-based near-infrared emitters (Nd, Yb) in the millisecond range through Cr (III) sensitization in discrete bimetallic edifices. Journal of the American Chemical Society.

[21] Harrison BS (2001). Near-infrared electroluminescence from conjugated polymer/lanthanide porphyrin blends. Applied Physics Letters.

[22] Wang H, Qian G, Wang M, Zhang J, Luo Y (2004). Enhanced luminescence of an erbium (iii) ion-association ternary complex with a near-infrared dye. The Journal of Physical Chemistry B.

[23] Zheng B, Ismagilov RF (2005). A microfluidic approach for screening submicroliter volumes against multiple reagents by using preformed arrays of nanoliter plugs in a three‐phase liquid/liquid/gas flow. Angewandte Chemie International Edition.

[24] Xiang H, Cheng J, Ma X, Zhou X, Chruma JJ (2013). Near-infrared phosphorescence: materials and applications. Chemical Society Reviews.

[25] Zhou L (2012). Simple, selective, and sensitive colorimetric and ratiometric fluorescence/phosphorescence probes for platinum (II) based on salen-type Schiff bases. RSC Advances.

[26] Zhou L (2012). Synthesis and photophysical properties of water-soluble sulfonato-Salen-type Schiff bases and their applications of fluorescence sensors for Cu2+ in water and living cells. Analytica chimica acta.

[27] Pschirer NG, Kohl C, Nolde F, Qu J, Müllen K (2006). Pentarylene‐and Hexarylenebis (dicarboximide) s: Near‐Infrared‐Absorbing Polyaromatic Dyes. Angewandte Chemie.

[28] Torelli S (2005). Tuning the Decay Time of Lanthanide‐Based Near Infrared Luminescence from Micro‐to Milliseconds through d → f Energy Transfer in Discrete Heterobimetallic Complexes. Chemistry–A European Journal.

[29] Shahroosvand, H. *et al*. Key role of ancillary ligands in imparting blue shift in electroluminescence wavelength in ruthenium polypyridyl light-emitting diodes. *New Journal of Chemistry* **38**, 5312–5323 (2014).

[30] Yap CC, Yahaya M, Salleh MM (2009). The effect of driving voltage on the electroluminescent property of a blend of poly (9-vinylcarbazole) and 2-(4-biphenylyl)-5-phenyl-1, 3, 4-oxadiazole. Current Applied Physics.

[31] Handy ES, Pal AJ, Rubner MF (1999). Solid-state light-emitting devices based on the tris-chelated ruthenium (II) complex. 2. Tris (bipyridyl) ruthenium (II) as a high-brightness emitter. Journal of the American Chemical Society.

[32] Ashford DL (2014). Controlling ground and excited state properties through ligand changes in ruthenium polypyridyl complexes. Inorganic chemistry.

[33] Shahroosvand H (2017). A ruthenium tetrazole complex-based high efficiency near infrared light electrochemical cell. Chemical Communications.

[34] Shahroosvand, H. *et al*. Dye-sensitized nanocrystalline ZnO solar cells based on Ruthenium (II) phendione complexes. *International Journal of Photoenergy* **2011**, 1–10 (2011).

[35] Chen Y (2010). Aggregation-induced emission of ruthenium (II) polypyridyl complex [Ru (bpy) 2 (pzta)] 2+. Inorganic Chemistry Communications.

[36] Brunner K (2004). Carbazole compounds as host materials for triplet emitters in organic light-emitting diodes: tuning the HOMO level without influencing the triplet energy in small molecules. Journal of the American Chemical Society.

[37] Slinker JD (2004). Efficient yellow electroluminescence from a single layer of a cyclometalated iridium complex. Journal of the american chemical society.

[38] Bolink HJ (2006). Stable single-layer light-emitting electrochemical cell using 4, 7-diphenyl-1, 10-phenanthroline-bis (2-phenylpyridine) iridium (III) hexafluorophosphate. Journal of the American Chemical Society.

[39] Bolink HJ, Cappelli L, Coronado E, Grätzel M, Nazeeruddin MK (2006). Efficient and stable solid-state light-emitting electrochemical cell using tris (4, 7-diphenyl-1, 10-phenanthroline) ruthenium (II) hexafluorophosphate. Journal of the American Chemical Society.

[40] Sun Y, Collins SN, Joyce LE, Turro C (2010). Unusual photophysical properties of a ruthenium (II) complex related to [Ru (bpy) 2 (dppz)] 2+. Inorganic chemistry.

[41] Tyson DS, Luman CR, Zhou X, Castellano FN (2001). New Ru (II) chromophores with extended excited-state lifetimes. Inorganic chemistry.

[42] Wilson GJ, Launikonis A, Sasse WH, Mau AW-H (1997). Excited-state processes in ruthenium (II) bipyridine complexes containing covalently bound arenes. The Journal of Physical Chemistry A.

[43] Tyson, D. S., Bialecki, J. & Castellano, F. N. Ruthenium (II) complex with a notably longexcited state lifetime. *Chemical Communications*, 2355-2356 (2000).

[44] Saha D, Das S, Mardanya S, Baitalik S (2012). Structural characterization and spectroelectrochemical, anion sensing and solvent dependence photophysical studies of a bimetallic Ru (ii) complex derived from 1, 3-di (1 H-imidazo [4, 5-f][1, 10] phenanthroline-2-yl) benzene. Dalton Transactions.

[45] Siebert R, Winter A, Schubert US, Dietzek B, Popp J (2011). The molecular mechanism of dual emission in terpyridine transition metal complexes—ultrafast investigations of photoinduced dynamics. Physical Chemistry Chemical Physics.

[46] Dixon IM, Lebon E, Sutra P, Igau A (2009). Luminescent ruthenium–polypyridine complexes & phosphorus ligands: anything but a simple story. Chemical Society Reviews.

[47] Ikeda N, Yoshimura A, Tsushima M, Ohno T (2000). Hopping and Annihilation of 3MLCT in the Crystalline Solid of [Ru (bpy) 3] X2 (X = Cl−, ClO4− and PF6−). The Journal of Physical Chemistry A.

[48] Fushimi T, Oda A, Ohkita H, Ito S (2004). Triplet Energy Migration in Layer-by-Layer Deposited Ultrathin Polymer Films Bearing Tris (2, 2 ‘-bipyridine) ruthenium (II) Moieties. The Journal of Physical Chemistry B.

[49] Creighton J, Ho P (2001). Introduction to chemical vapor deposition (CVD). Chemical vapor deposition.

[50] Dreyse P (2013). Effect of free rotation in polypyridinic ligands of Ru (II) complexes applied in light-emitting electrochemical cells. Dalton Transactions.

[51] Gao FG, Bard AJ (2000). Solid-state organic light-emitting diodes based on tris (2, 2’-bipyridine) ruthenium (II) complexes. Journal of the American Chemical Society.

