# Supplementary material for: A near infrared light emitting electrochemical cell with a 2.3 V turn-on voltage
Source: Sci Rep. 2019 Jan 18;9:228. doi: 10.1038/s41598-018-36420-1 (PMC6338728; doi:10.1038/s41598-018-36420-1)
Supplement: Supplementary file 1 — ESI [file 41598_2018_36420_MOESM1_ESM.docx]

Supplemenatry Information for

A near infrared light emitting electrochemical cell with a 2.3 V turn-on voltage

Babak Nemati Bideh, Hashem Shahroosvand, Ahmad Sousaraei, Juan Cabanillas-Gonzalez

**Materials and methods.**

All reagents were obtained commercially and used as received. All reaction were carried out under inert atmosphere (N_2_) with standard Schlenk technique. The ^1^H and ^13^C NMR spectra were measured on a Bruker 250 MHz instrument with CDCl_3_, D6-DMSO and tetramethylsilane (TMS) as solvent and internal standard, respectively. FT-IR spectra were recorded on a Perkin-Elmer 597 spectrometer and measured on KBr pellets. Elemental analyses were performed on Elementar Vario EL CHN elemental analyzer. Cyclic voltammetry (CV) was carried out in nitrogen purged acetonitrile solution under a dry N2 atmosphere at room temperature by using a SAMA500 potentiostat electrochemical analyzerwith conventional three electrode cell, a Pt disk as the working electrode, a Pt wire as the counter electrode, and Ag/AgCl as the reference electrode and using 0.10 M tetrabutyl ammonium perchlorate (TBAP) as the supporting electrolyte. For the parameter calculation the following relation were used: scan rate, 50 mV s-1; formal potential E_ο_^′^ = (Epa +/Epc)/2 where E_pa_ and E_pc_ are anodic and cathodic peak potentials, respectively; ∆Ep is the peak-to-peak separation. The oxidation (E_ox_) were used to calculate the HOMO energy levels and energy gap (E_gap_) using the equations E_HOMO_ =[-e(E_ox_(vs.Ag/AgCl) – E_1/2_(FC/Fc^+^vs. Ag/AgCl))] – 4.8 eV wich is the reduction potential of ferrocene which was found to be 0.43 V. The LUMO energy level was determined as E_LUMO_ = E_HOMO_ + E_0-0_ eV, (E_0-0_: optical band gap derived from the absorption onset in UV/Vis spectra). E_gap_= -E_HOMO_ – E_LUMO_[1-4]. UV–visible absorption spectra was recorded on a Ultrospec3100 pro spectrophotometer in acetonitrile solutions. Photoluminescence (PL) spectra of complexes in degassed solutions at 298 K and neat films were recorded using Varian-Cary Eclipse flourescence spectrophotometer and AvaSpec-125 spectrophotometer, respectively. The PLQY (PL quantum yields) were calculated against [Ru(bpy)_3_]^2+^ in degassed acetonitrile solution at 298 K as a standard ($\emptyset_{std}=0.095$[5]) using the following equation:

$$\emptyset_{unk}=\emptyset_{std}.(\frac{I_{unk}/A_{unk}}{A_{std}/I_{std}}).{(\frac{\eta_{unk}}{\eta_{std}})}^{2}$$

In this equation,Φ_unk_ is phosphorescence quantum yields of complexes, I_unk_ and I_std_ are the integrated areas of the corrected PL spectra and standard, respectively, A_unk_ and A_std_ are the absorbances of the ruthenium complexes and the standard at the excitation wavelength (λ_exc_= 460 nm), and η_unk_ and η_std_ are the indexes of refraction of the respective solvents (taken to be equal to the neat solvents in both cases). Thin films of cationic ruthenium complexes for study of solid emission were obtained by drop cast from a spectrophotometric grade acetonitrile solution on a glass support with a thickness of about 90 nm. After evaporation of the solvent in air, the films were dried overnight under vacuum at room temperature.

Time-resolved PL measurements in solutions and films were performed with a HydraHarp Picoquant multichannel time correlator. Excitationwas provided using a 405 Sepia picosecond diode laser delivering

pulses of less than 130 ps with 2 MHz repetition rate. Single wavelength detection was conducted with a thermo-electrically cooled Hamamatsu photomultiplier coupled to a 0.5mSP-2558 Princeton Instruments (Acton Research) spectrometer equipped with a 600 lines/mm grating.

**LEEC fabrication and measurement :**LEEC devices were fabricated on ITO-coated glass substrates with a sheet resistance of 20 Ω/square. After being sufficiently cleaned by soaking in ultrasonicated isopropanol, acetone and deionized water, they were dried in the oven at 110 °C for 2h. The devices were prepared by spin-coating a thin layer of each complex (F1, F2, F3) on top of an ITO glass substrate from a 6% (w/v) acetonitrile solutionfor F1, F2 and 6% (w/v) acetonitrile (70%) + DMF (30%) solution for F3 at RT. All solution and film preparation were performed under ambient conditions. The thicknesses of the films were ~126 nm, measured with FE-SEM.


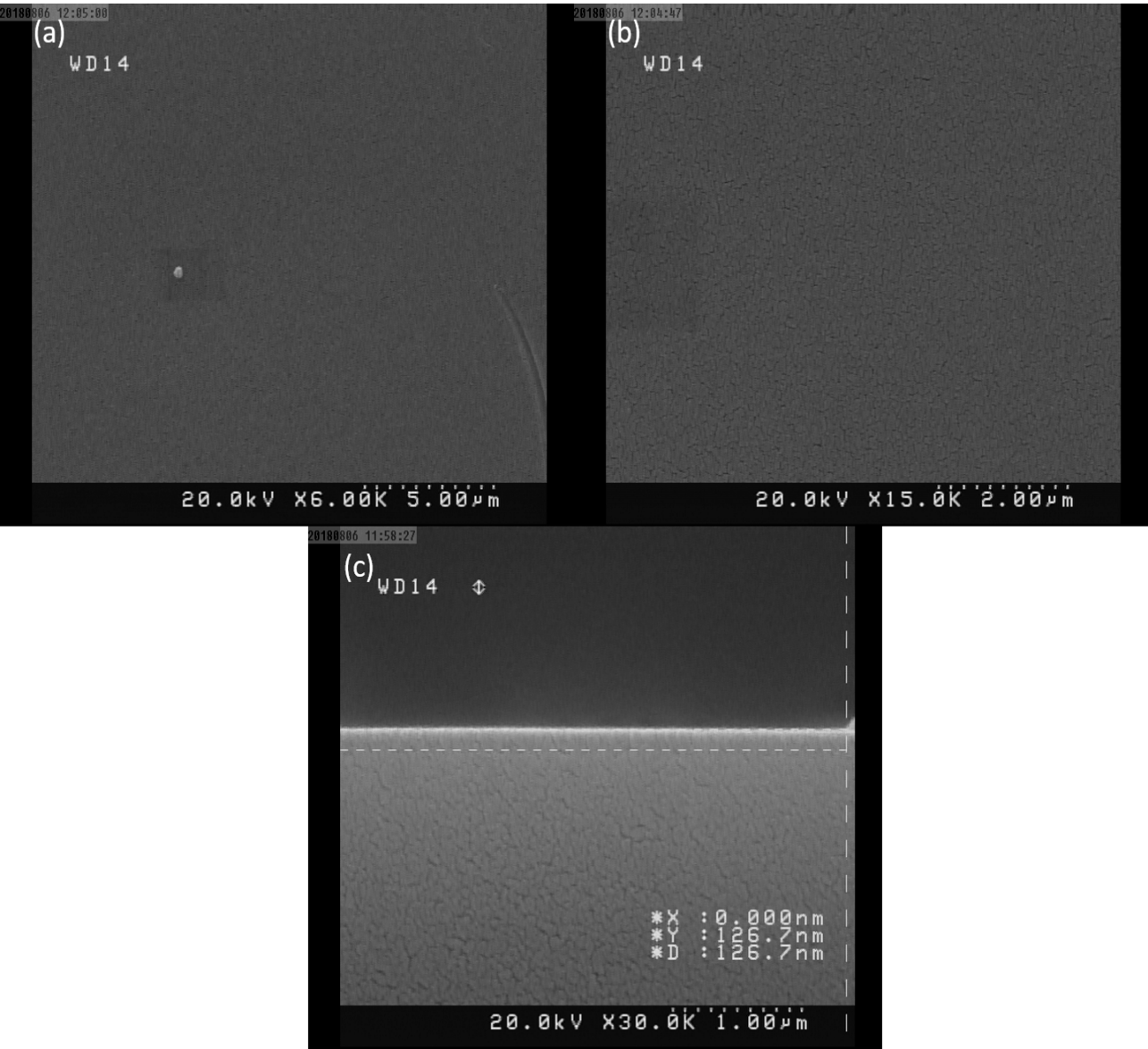


Figure S1. The top view (a,b) and cross section of FE-SEM of complex (F3) deposited on the glass. The image shows a uniform layer of F3 on top of ITO without cracking and pinhole defects with thickness of 126 nm

After spincoating, the thin films were annealed at 90 °C in inert atmosphere for 14h. A Ga: In (75.5:24.5 wt %, mp 15.7 °C) eutectic as cathode (ca. 3.5 mm diameter) was printed on the top of the active layer at room temperature by using a special syringe and then connected via a thin copper wire inserted into the Ga:In contact. Finally it was sealed with an epoxy cement. All EL measurement were carried out in air. Current density-voltage-luminance (J-V-L) characteristics and electroluminescence (EL) spectra of LEEC devices were measured using an AvaSpec-125 spectrophotometer, a SAMA500 electroanalayzer system and a Photo Research PR-650 spectroradiometer.

**Quantum chemical calculations:** The molecular and electronic structure calculations were performed with density functional theory (DFT) using the Gaussian 03(G03) software package. The B3LYP functional with the LANL2DZ basis set was carried out [6]. All geometry optimizations were performed in either C1 or C2 symmetry with subsequent frequency analysis to show that the structures are at the local minima on the potential energy surface. The electronic orbitals were visualized using Gauss View 3.0.

**Synthesis and characterization:** The compounds cis-[Ru(dmbpy)_2_Cl_2_].2H_2_O, cis-[Ru(bpy)_2_Cl_2_].2H_2_O, cis-[Ru(phen)_2_Cl_2_].2H_2_O and 1,10-phenanthroline-5,6-dione (phendione) were synthesized according to literature methods [1-10].

**Synthesis of (E)-1-(4-methoxyphenyl)-2-styryl-1H-imidazo[4,5-f][1,10]phenanthroline (****MPSIP) :** 1,10-phenanthroline-5,6-dione (0.212 g, 1.0 mmol), trans-Cinnamaldehyde (0.132 g, 1.0 mmol), p-Anisidine (0.123 g, 1 mmol) and ammonium acetate (770 mg, 10 mmol, excess) was refluxed in glacial acetic acid (8 mL) for 24 h under a N_2_ atmosphere then cooled to room temperature and diluted with 40 mL deionized water. The suspension mixture neutralizedwith a concentrated aqueous ammonia solution and extracted with 50 mL of chloroform and then removed the solvent by rotary evaporation and the residue was washed with cool ethanol and acetone. The recrystallization from CH_2_Cl_2_–acetone was repeated one more time to give the product as a grey solid. Yield: 41%. mp. 265°C, Anal. calcd. For C_28_H_20_N_4_O (%): C, 78.49 ; H, 4.70; N, 13.08. Found (%): C, 78.48 ; H, 4.70; N, 13.09. IR (KBr): $\tilde{\nu}=$ 3064 (C-H aromatic), 2963 (C-H aliphatic), 1621 (C=C), 1609 (C=N) cm^-1^ .^1^HNMR (250 MHz, CDCl_3_): 9.01-9.17(m, 2H), 9.02(d, 1H), 7.96 (d, 1H), 7.74 (m, 1H), 7.48-7.09 (m, 11H), 6.71 (d, 1H),4.01 (s, 3H). ^13^CNMR (62 MHz, CDCl_3_): 160.82, 152.55, 151.44, 148.89, 147.78, 136.00, 133.25, 130.49, 129.57, 128.87, 128.73, 127.87, 127.16, 124.01, 123.38, 122.18, 119.61, 115.64, 113.37, 55.7.

**General procedure for synthesis of [Ru(N^N)_2_(MPSIP)](ClO_4_)_2_:** A mixture of cis-[Ru(N^N)_2_Cl_2_].2H_2_O (0.1 mmol) and MPSIP (0.428 gr , 1 mmol) was degassed by N_2_ and heated at 125 °C in ethylene glycol (5 ml) for 18 h under N_2_ to give a clear red solution. Upon cooling, saturated aqueous sodium perchlorate solution was added under vigorous stirring and the orange solid was collected and washed several times with deionized water to remove traces of salts.The crude solid product was purified by column chromatography on alumina with acetonitrile-toluene (3/1, v/v) as an eluent. The mainly red band was collected. The solvent was removed under reduced pressure and red solid were obtained.

**CAUTION:** Perchlorate salts of metal complexes are potentially explosive, and only small amounts of the material should be prepared and handled with great care!

**[Ru(bpy)_2_(MPSIP)](ClO_4_)_2_ (F1).** Yield: 69% . Anal. calcd. For C_48_H_36_Cl_2_N_8_O_9_Ru (%): C, 55.39; H, 3.49; N, 10.77. Found (%): C, 55.38; H, 3.50; N, 10.78. (MW, 1040). IR (KBr): $\tilde{\nu}=$ 3076 (C-H aromatic), 2961 (C-H aliphatic), 1619 (C=C), 1604 (C=N), 1084 (ClO_4_) cm^-1^. ^1^HNMR (250 MHz, D_6_-DMSO): 9.14 (d, 1H), 8.79-8.99 (m, 4H), 8.07-8.79 (m, 8H), 7.08-7.99 (m, 4H), 8.33 (d, 1H), 7.55-7.85 (m, 8H), 7.28-7.36 (m, 6H),6.79 (d, 1H), 3.93 (s, 3H).^13^CNMR (62 MHz, D_6_-DMSO):161.08, 157.18, 157.11, 156.99, 153.47, 152.33, 151.91,150.57, 145.86, 145.66, 138.46, 138.33, 137.30, 135.61, 134.41, 131.01, 130.62, 130.09, 129.47, 128.26, 127.79, 126.27, 125.72, 124.92, 121.69, 116.41, 113.53, 56.16.

**[Ru (dmbpy)_2_(MPSIP)](ClO_4_)_2_ (F2).**Yield: 72%. Anal. calcd. For C_52_H_44_Cl_2_N_8_O_9_Ru (%): C, 56.94; H, 4.04; N, 10.22. Found (%): C, 56.94; H, 4.05; N, 10.23. (MW, 1096). IR (KBr): $\tilde{\nu}=$ 3078 (C-H aromatic), 2941 (C-H aliphatic), 1613 (C=C), 1605 (C=N), 1081 (ClO_4_) cm^-1^. ^1^HNMR (250 MHz, D_6_-DMSO): 9.08 (d, 1H), 8.91 (d, 1H), 8.66 (m, 4H), 8.12 (d, 1H), 7.93(m, 3H), 7.66 (m, 4H), 7.49 (s, 4H),7.29-7.51 (m, 7H), 7.16 (b, 2H), 6.70 (d, 1H), 3.91 (s, 3H), 2.51 (s, 6H, 4^’^-CH_3_), 2.43 (s, 6H, 4-CH_3_). ^13^CNMR (62 MHz, D_6_-DMSO):161.17, 156.57, 153.27, 150.78, 150.13, 150.02, 146.05, 145.88,137.24, 136.91 , 135.59, 130.58, 130.05, 129.89, 129.43, 128.99, 128.07, 127.85, 127.72, 127.48, 126.10, 125.62, 125.44, 121.60, 116.31, 113.49, 56.12, 21.07.

**Ru (phen)_2_(MPSIP)](ClO_4_)_2_ (F3).** Yield: 81%. Anal. calcd. For C_52_H_36_Cl_2_N_8_O_9_Ru (%): C, 57.36; H, 3.33; N, 10.29. Found (%): C, 57.35; H, 3.34; N, 10.30. (MW, 1089). IR (KBr): $\tilde{\nu}=$ 3069 (C-H aromatic), 2954 (C-H aliphatic), 1603 (C=C), 1595 (C=N), 1083 (ClO_4_) cm^-1^. ^1^HNMR (250 MHz, D_6_-DMSO): 9.06 (d, 1H), 8.73 (d, 4H), 8.35 (d, 4H), 8.13 (d, 2H), 8.07(m, 2H),7.94 (m, 2H), 8.78-7.64 (m, 7H), 7.52 (m, 4H), 7.32 (m, 6H), 6.78 (d, 1H), 3.91 (s, 3H). ^13^CNMR (62 MHz, D_6_-DMSO):161.19, 153.30, 153.21, 153.01, 151.44, 150.61, 147.67, 147.58, 147.52, 146.28, 146.10, 138.30, 137.31, 136.95, 135.60, 130.92, 130.58, 130.06, 129.46, 128.52, 127.86, 127.76, 127.03, 126.77, 126.11, 125.66, 121.61, 116.41, 113.52, 56.14.


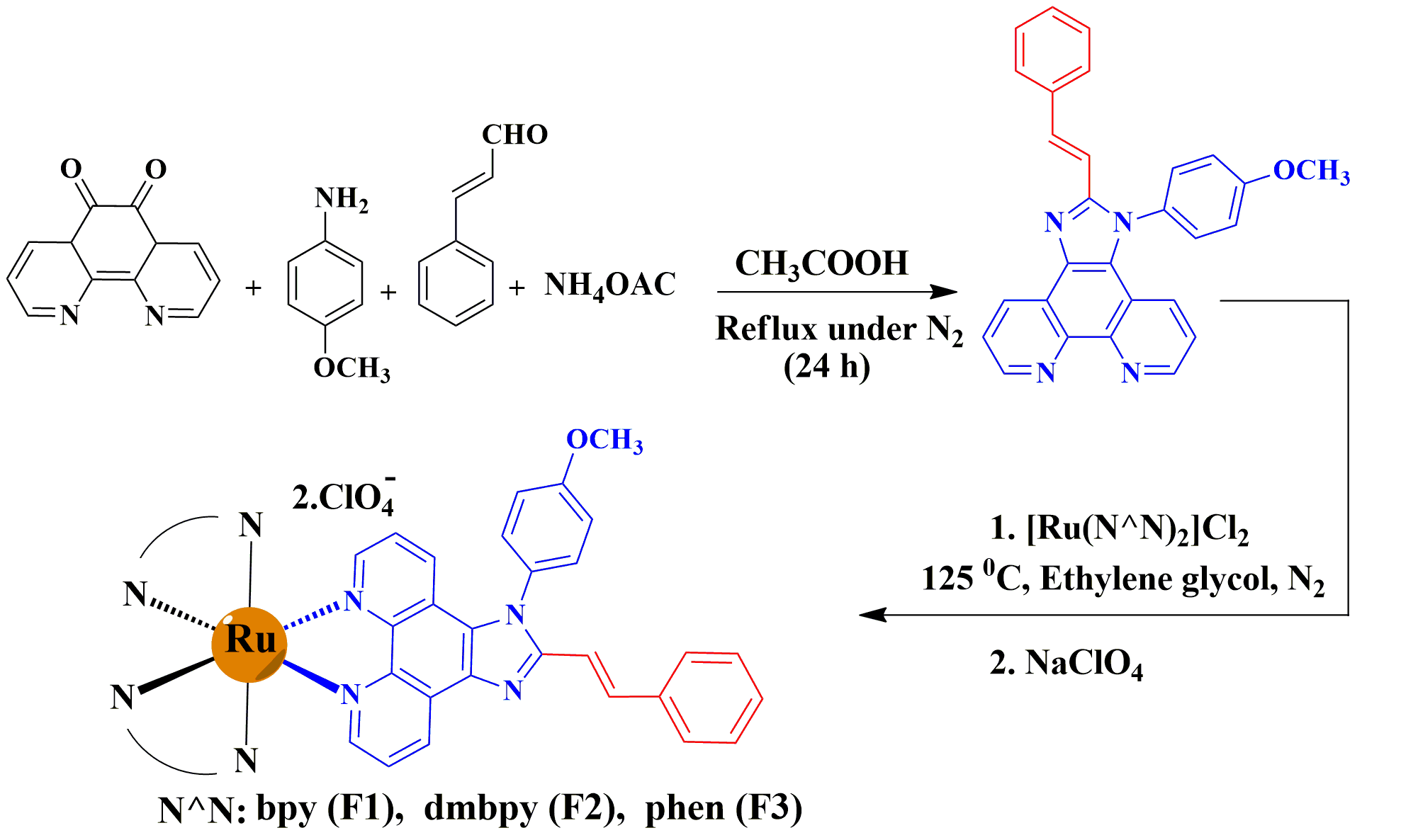


**Scheme S1.** Synthesis of phenanthroimidazole ligand (MPSIP) and their ruthenium(II) complexes


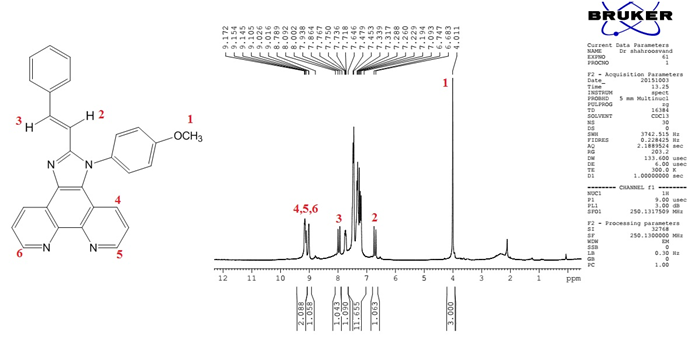


**Figure S2.**^1^HNMR of MPSIPin CDCl_3_


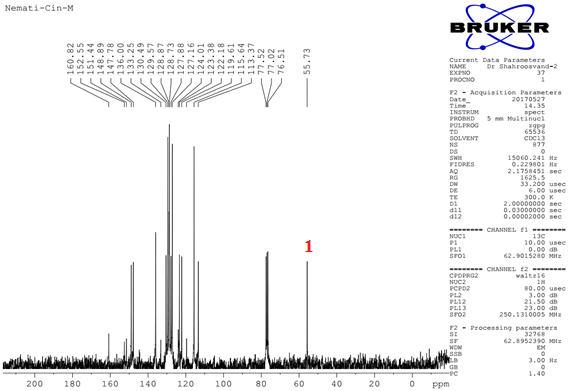


**Figure S3.**^13^CNMR of MPSIP in CDCl_3_


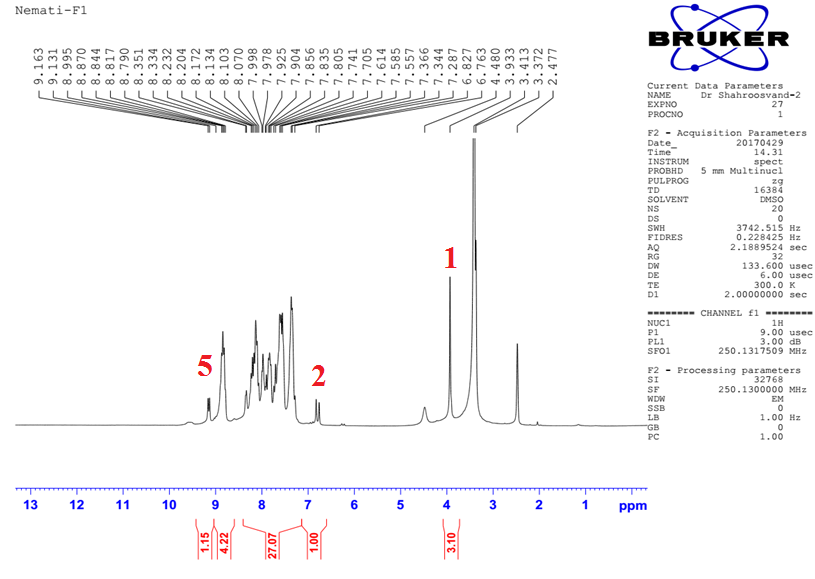


**Figure S4.**^1^HNMR of F1 in D_6_-DMSO as solvent

**
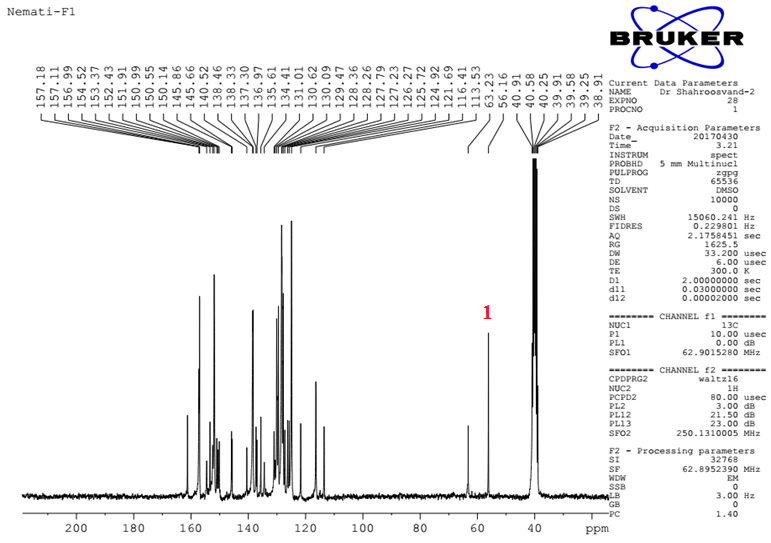
**

**Figure S5.**^13^CNMR of F1 in D_6_-DMSO as solvent

**
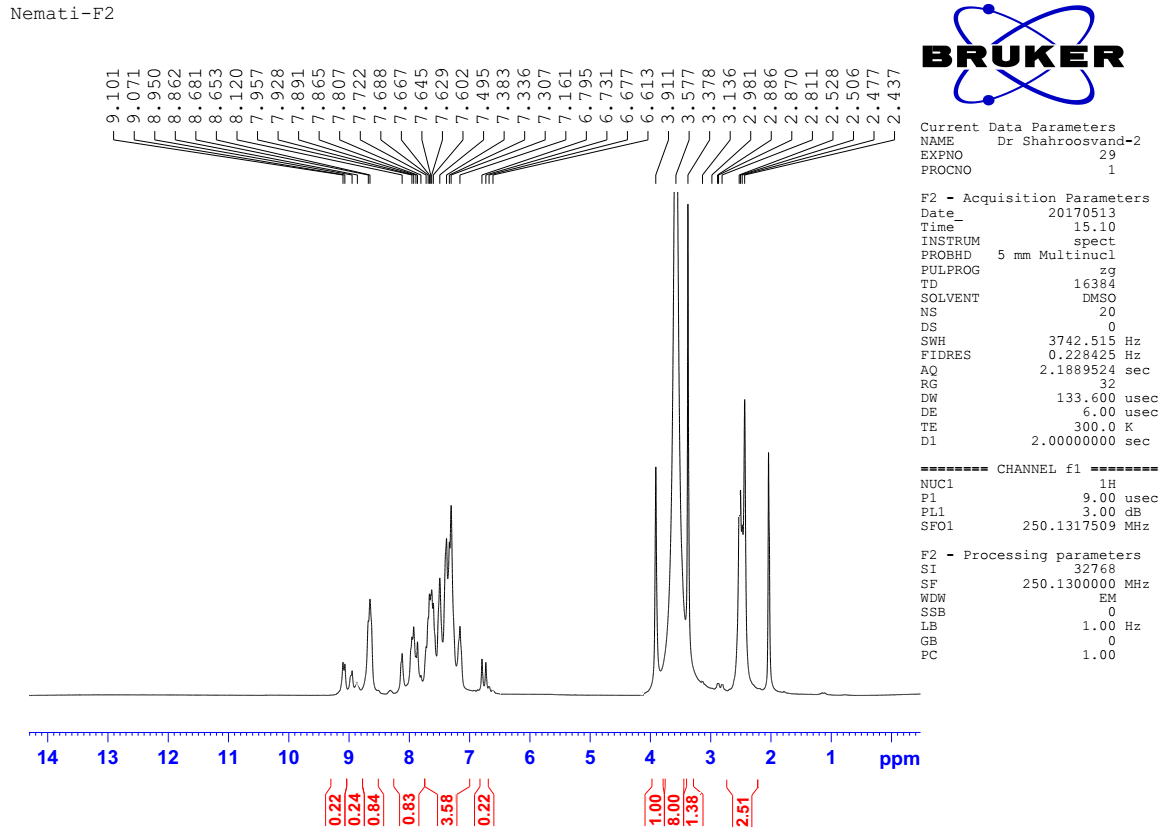
**

**Figure S6.**^1^HNMR of F2 in D_6_-DMSO as solvent

**
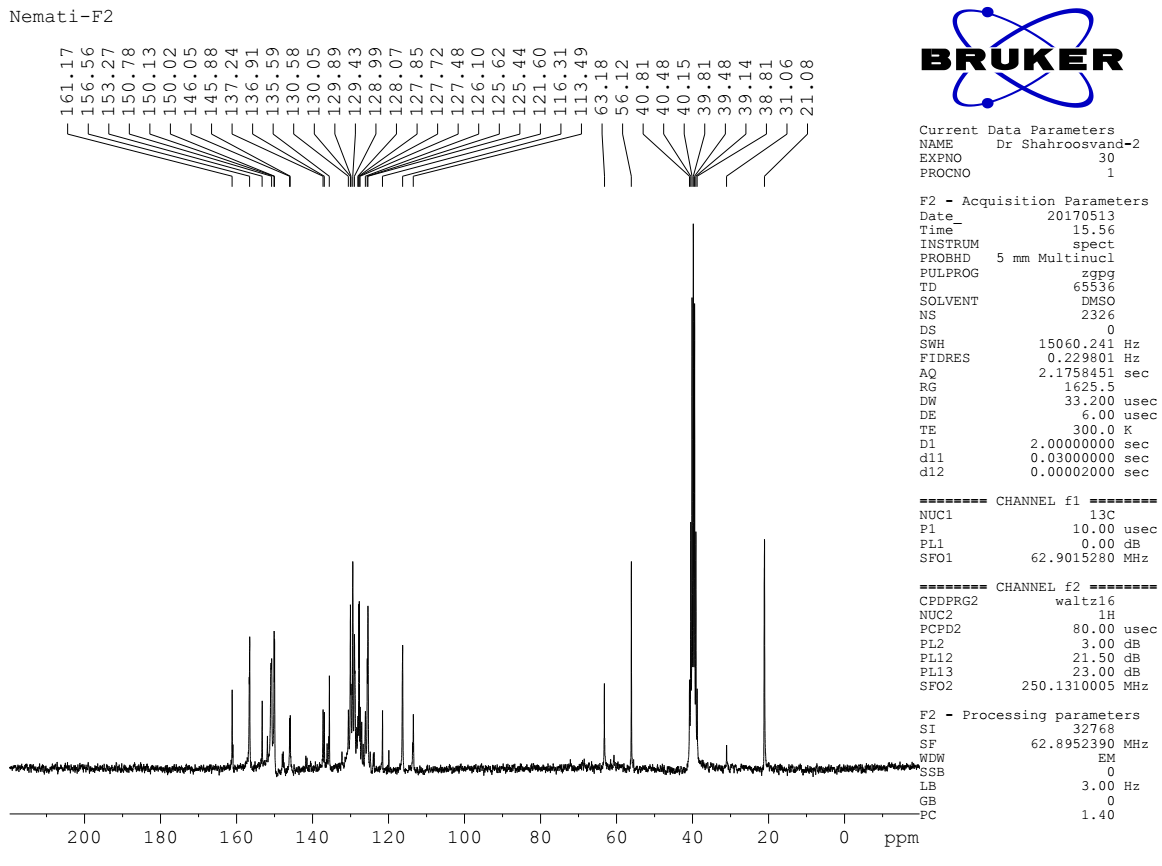
**

**Figure S7.**^13^CNMR of F2 in D_6_-DMSO as solvent

**
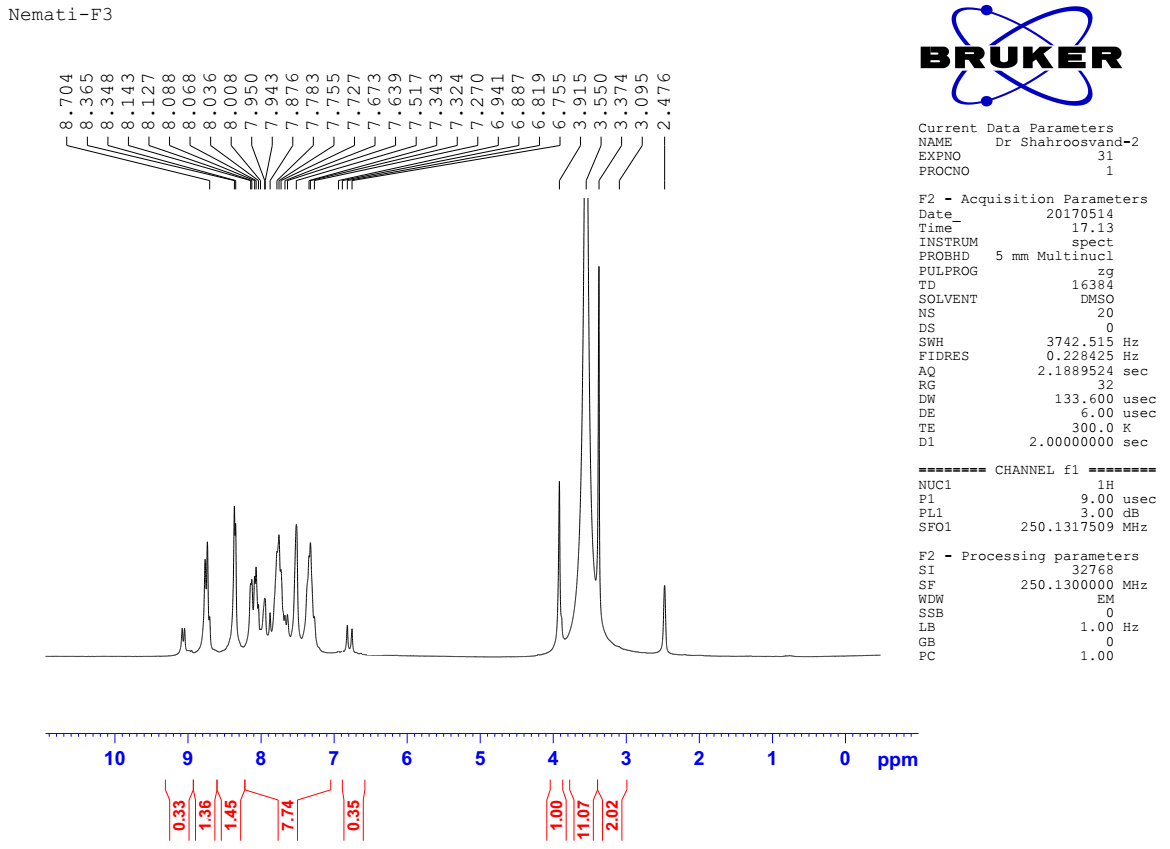
**

**Figure S8.**^1^HNMR of F3 in D_6_-DMSO as solvent

**
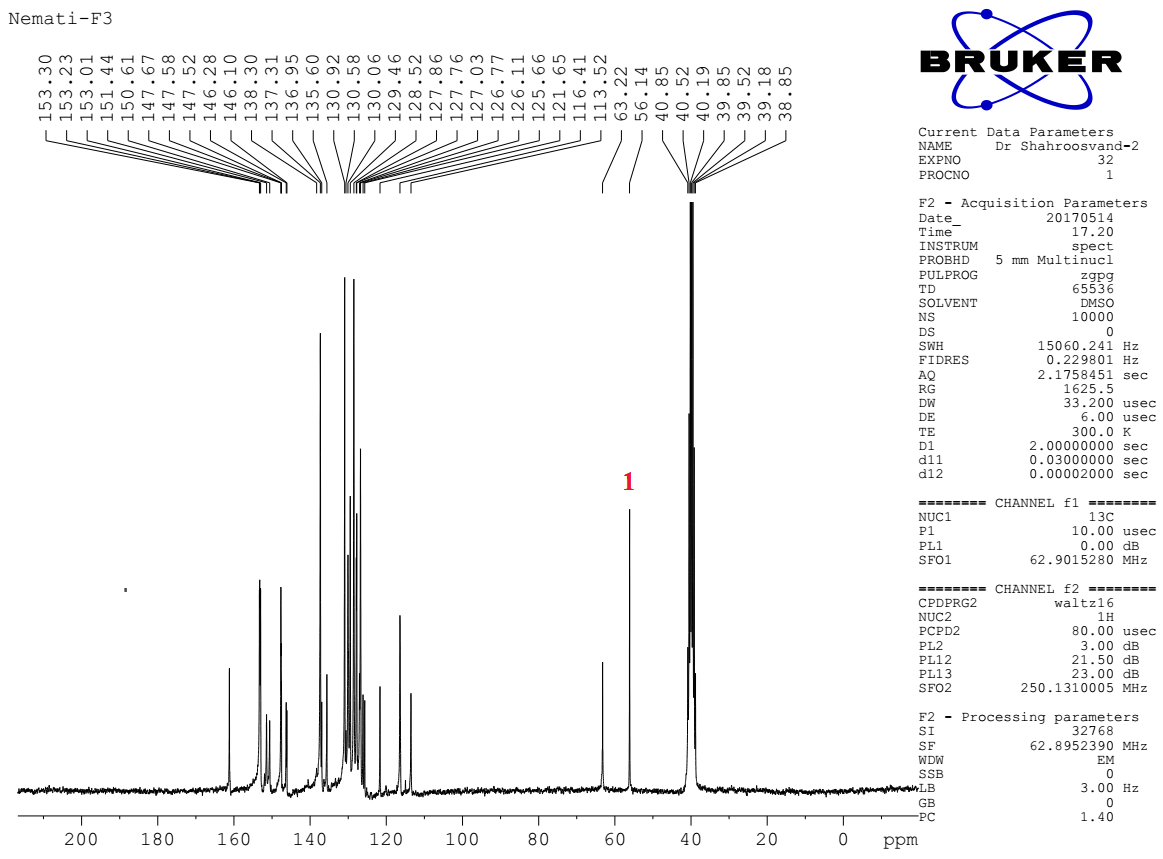
**

**Figure S9.**^13^CNMR of F3 in D_6_-DMSO as solvent


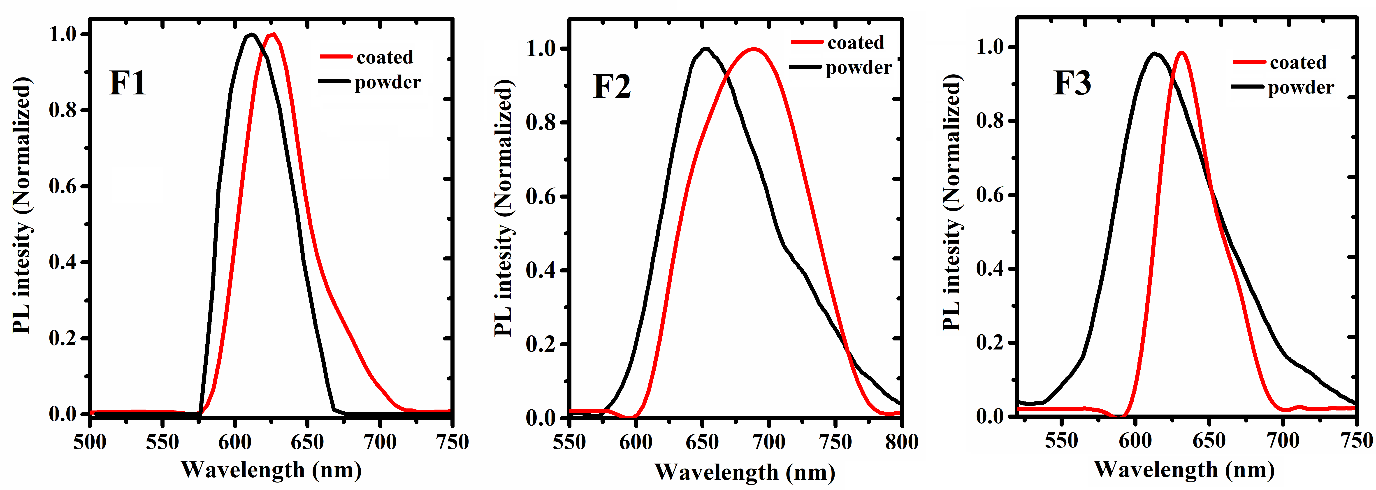


**Figure S10**. The PL of F1-3 in solid state (powder and coated on ITO)-The red shift of solid PL in crystalline (coated) respect to the powder form, exhibited aggregation of complexes in solid phase.

**
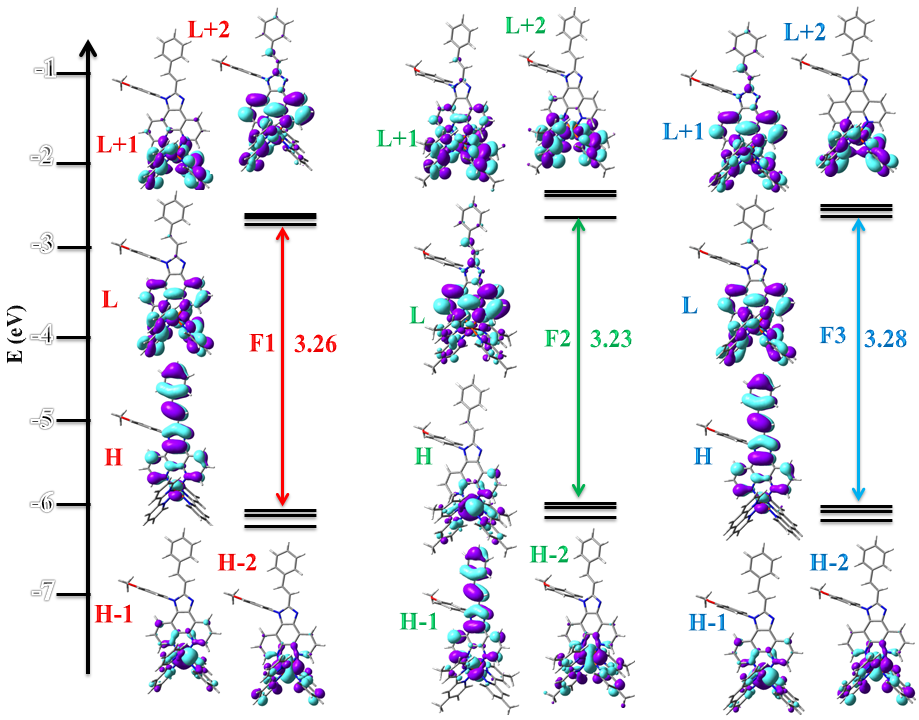
**

**Figure S11**. Isosurfaces for the HOMO and LUMO of ruthenium complexes (F1-F3) obtained from DFT method through LANL2DZ basis set.


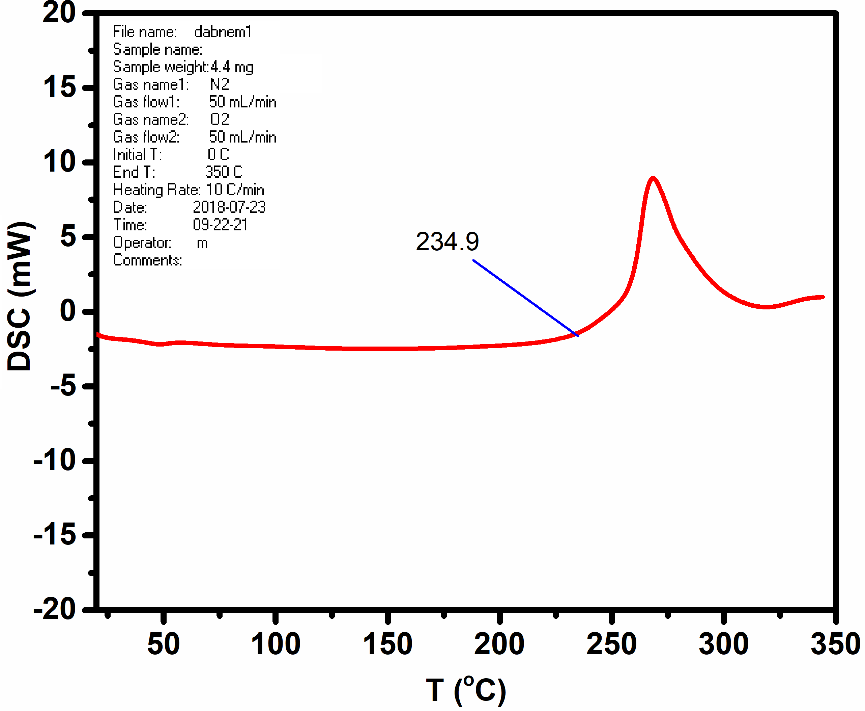


**Figure S12**. The DSC curve of complex F3 shows the good thermal stability of emitter with Tg = 234.9 ^o^C, Tc = 275 ^o^C and melting point higher than 350 ^o^C

**Table S1.** A review on the Turn on voltage value for OLED based on some important transition metal complexes by ancillary ligand substitution

| **Complex** | **TP** | **PL_max_(nm)** | **EL_max_ (nm)** | **V_on_ (V)** | **Ref.** |  |
| --- | --- | --- | --- | --- | --- | --- |
|  | ITO\|PEDOT:PSS\|**Com**:PVK\|BCP\|LiF:Al | 558 | 567 | 8 | 17 |  |
|  | ITO\| **Com** Ag | 491,  520 | 550 | 2.5 | 18 |  |
|  | ITO\|PEDOT:PSS\|**Com**:PVK \|Ca:Al | 564 | 553 | 6 | 19 |  |
|  | ITO\|**Com**:PVK\|Al | 580 | 600 | 8 | 20 |  |
|  | ITO\| **Com**:PVCz:TPD\|BCP\|Alq_3_\|Mg:Ag | - | 380,  540 | 6 | 21 |  |
|  | ITO **Com** \|BCP\|Alq3\|Mg:Ag | - | - | 6- | 21 |  |
|  | ITO\|NPB\| **Com** or **Com**:CBP\|BCP\|Alq_3_\|LiF:Al | 540  540 | 540  540 | 3.5  4 | 22 |  |
|  | ITO\|NPB\| **Com** or **Com**:CBP\|BCP\|Alq_3_\|LiF:Al | 528  530 | 528  530 | 4  4 | 22 |  |
|  | ITO\|NPB\| **Com** or **Com**:CBP\|BCP\|Alq_3_\|LiF:Al | 535  532 | 535  532 | 4  3.5 | 22 |  |
|  | ITO\|NPB **Com**:CBP\|BCP\|Alq_3_\|LiF:Al | 532 | 532 | 4 | 22 |  |
|  | ITO\|NPB\| **Com**:CBP\|BCP\|Alq_3_\|LiF:Al | 558  560  558  546 | 558  560  558  548 | 4  4  4  4 | 22 |  |
|  | ITO\|PEDOT:PSS\|PVK **Com** \|Ca:Al | 450, 478, 510 | 488 | 3 | 23 |  |
|  | ITO\|NPB\|r **Com** \|TPBI\|LiF:Al | 530  558 | -  - | 3.5  3.7 | 24 |  |
|  | ITO\|NPB\|**Com**:CBP\|TPBI\|LiF:Al | -  -  - | 491  490  490 | 3.3  3.2  3.0 | 25 |  |
|  | ITO\|NPB\|**Com**:CBP\|TPBI\|LiF:Al | 576,  605  579,  610 | 565  - | 5.0  - | 26 |  |
|  | | ITO\|**Com**:PEO\|Au | 570 | 640 | 3.6 | 27 |
|  | | ITO\|**Com**:PEO\|Au | 550 | 600 | 5.4 | 27 |
|  | | ITO\|NPB\| **Com**:CBP\|BCP\|Alq_3_\|Liq:Al | - | 627 | 4.4 | 29 |

|  | ITO\| **Com** :PEO\|Au | 550 | 600 | 5.4 | 27 |
| --- | --- | --- | --- | --- | --- |
|  | ITO\|PEDOT:PSS\| **Com** :PVCz:PBD\|CsF:Al | 635  634  638 | 637  636  639 | -  -  - | 28 |
|  | ITO\|NPB\| **Com** :CBP\|BCP\|Alq_3_\|Liq:Al | - | 627 | 4.4 | 29 |
|  | ITO\|NPB\| **Com** :CBP\|BCP\|Alq_3_\|Liq:Al | - | 625 | 4.2 | 29 |
|  | ITO\|NPB\| **Com** :CBP\|BCP\|Alq_3_\|Liq:Al | - | 578 | 3.6 | 29 |
|  | ITO\|NPB\| **Com** :CBP\|BCP\|Alq_3_\|Liq:Al | - | 607 | 3 | 29 |
|  | ITO\|NPB\| **Com** :CBP\|Bphen\|Liq:Al | 603 | - | 3.5 | 30 |
|  | ITO\|NPB\| **Com** :CBP\|Bphen\|Liq:Al | - | 636 | 3.5 | 30 |
|  | ITO\|NPB\| **Com** :CBP\|Bphen\|Liq:Al | - | 624 | 3.5 | 30 |
|  | ITO\|NPB\| **Com** :CBP\|Bphen\|Liq:Al | - | 614 | 3.5 | 30 |
|  | ITO\|NPB\| **Com** :CBP\|Bphen\|Liq:Al | - | 593 | 3.5 | 30 |
|  | ITO\|NPB\| **Com** :CBP\|Bphen\|Liq:Al | - | 575 | 3.5 | 30 |
|  | ITO\|PEDOT:PSS **Com** :PVK\|TPBI\|LiF:Al | -  - | 581  589 | 8.3  10.75 | 31 |
|  | ITO\|PEDOT:PSS **Com** :PVK\|TPBI\|LiF:Al | -  - | 550  557 | 7.6  7.8 | 31 |
|  | ITO\|PEDOT:PSS\|Ir:CBP:TCTA\|TmPyPb\|LiF\|Al | -  - | 608  624 | 4.2  5.4 | 32 |
|  |  | - | 616 | 4.9 | 32 |

|  | | ITO\|NPB**Com**:CBP\|Bphen\|Liq:Al | | -  -  -  -  -  - | 606  623  627  619  578  589 | | | | 3.5  4.0  4.5  4.5  4.5  4.0 | | | 33 | | |
| --- | --- | --- | --- | --- | --- | --- | --- | --- | --- | --- | --- | --- | --- | --- |
|  | | ITO\|NPB\| **Com**:CBP\|BCP\|Alq_3_\|LiF:Al | | -  -  -  - | 552  532  564  560 | | | | 3.8  3.9  3.8  3.3 | | | 34 | | |
|  | | ITO\|MoO_3_\|NPB\|TCTA\|TCTA **Com** \|TPBi\|LiF\|Al | | | 485, 512  499, 525 | 492  500 | | | 2.8  3 | | | | 35 |  |
|  | | ITO\|PEDOT:PSS\| **Com**:PVK:OXD-7:UGH3\|TmPyPB\|LiF:Al | | | 475,  494  466,  489 | 472,  502  478,  502 | | | 5  5.5 | | | | 35 |  |
|  | | ITO\|TAPC\|**Com**:mCP\|TPBi\|LiF:Al | | | 564  510 | 526  489 | | | 3.1  3.2 | | | | 36 |  |
|  | | ITO\|TAPC\| **Com**:SimCP2\|TPBi\|LiF:Al | | | 541  567  514  564  487  548 | -  534 503 521 480 543 | | | -  3.5  3.6  3.9  3.7  3.6 | | | | 37 |  |
|  | | ITO\|TAPC\| **Com**: mCP\|TPBi\|LiF:Al | | | -  -  - | 497  497  495 | | | 3.3  3.3  3.3 | | | | 38 |  |
|  | | ITO\|TAPC\| **Com**:SimCP2\|TPBi\|LiF:Al | | | 551  549  531 | 534  512  509 | | | 3.5  3.5  3.6 | | | | 39 |  |
|  | | ITO\|PEDOT:PSS\| **Com**:mCP\|OXD-7\|LiF:Al | | | 464, 486  456, 478 | -  - | | | 14  14 | | | | 40 |  |
|  | | ITO\|PEDOT:PSS\| **Com**:BMIMPF_6_\|Al | | | 551  501 | 556  497 | | | 4  4 | | | | 41 |  |
|  | | ITO\|PEDOT:PSS\| **Com**:BMIMPF_6_\|Al | | | 582  526, 550 | 588  552, 528 | | | 3.5  3.5 | | | | 41 |  |
|  | | ITO\|PEDOT:PSS\| **Com**:BMIMPF_6_\|Al | | | 628, 640 | 650 | | | 3.5 | | | | 41 |  |
|  | | ITO\|PEDOT:PSS\| **Com**:BMIMPF_6_\|Al | | | 492, 523 | 524, 497 | | | 5 | | | | 41 |  |
|  | | ITO\|PEDOT:PSS\| **Com**:BMIMPF_6_\|Al | | |  | 497, 590 | | | 3.5 | | | | ^41^ |  |
|  | | ITO\|PEDOT:PSS\| **Com**:PVK:OXD-7\|TPBi\|Cs_2_CO_3_\|Al | | | 508  482 | 506  482 | | | 4.9  6 | | | | 42 |  |
|  | | ITO\|MoO_x_\|TCTA\|mCP\|BCPO **Com** \|LiF\|Al | | | - | 451 | | | 3.2 | | | | 43 |  |
|  | | ITO\|MoO_x_\|TCTA\|mCP\|BCPO: **Com** \|LiF\|Al | | | - | 569 | | | 3.8 | | | | 43 |  |
|  | | ITO\|PEDOT:PSS\| **Com**:PVK:OXD-7\|TPBI\|CsF:Al | | | 487, 513 | - | | | 3.4 | | | | 44 |  |
|  | | ITO\|Plexcore\|TAPC\| **Com**:26DCzPPy\|BmPyPB\|Cs_2_CO_3_\|Al | | | 467, 490 | - | | | 3.1 | | | | 44 |  |
|  | | ITO\|TAPC\| **Com**:SimCP2\|TPBi\|LiF:Al | | | 566  566  556  564 | 526  534  520  519 | | | 3.3  3.3  3.3  3.5 | | | | 45 |  |
|  | | ITO\|PEDOT:PSS\| **Com**:PVK:OXD-7\|TPBI\|Cs_2_CO_3_\|Al | | | 516  476 | 498  478 | | | 6  6.2 | | | | 46 |  |
|  | | ITO\|PEDOT:PSS\|PVK\| **Com**:mCP:OXD-7\|B3PYMPM\|Ca\|Al | | | 519  507 | 520  501 | | | 8.8  8.1 | | | | 47 |  |
|  | | ITO\|PEDOT:PSS\| **Com** \|BCP\|Alq_3_\|LiF:Al | | | -  -  -  - | -  -  -  - | | | 7.8  8.0  8.1  8.1 | | | | 48 |  |
|  | | ITO\|MPMP\| **Com** \|DPA\|Alq_3_\|LiF:Al | | | -  -  - | 540, 580  540  560 | | | -  19.5  - | | | | 49 |  |
|  | | ITO\|CuPc\| **Com** \|Ca:Al | | | 550, 590, 668 | 538, 578 | | | 6 | | | | 50 |  |
|  | | ITO\|NPB\| **Com** \|TPBI\|LiF:Al | | | -  -  -  -  -  - | 537  53  542  540  -  - | | | 3.5  6.3  4.7  5.6  -  - | | | | 51 |  |
|  | | ITO\|PEDOT:PSS **Com**: CBP\|BCP\|Alq_3_\|LiF:Al | | | - | 540 | | | 8 | | | | 52 |  |
|  | | ITO\|PEDOT:PSS\| **Com**: CBP\|BCP\|Alq_3_\|LiF:Al | | | -  -  -  -  -  - | -  494  531  512  -  - | | | -  8.6  7.7  3.3  -  - | | | | 53 |  |
|  | | ITO\|PEDOT:PSS\| **Com**: CBP\|BCP\|Alq_3_\|LiF:Al | | | - | 547 | | | 12.6 | | | | 53 |  |
|  | | ITO\| **Com** \|BATH\|Alq_3_\|Yb | | | 489, 521  488, 520  491, 524 | -  -  - | | | 10.0  9.0  11.5 | | | | 54 |  |
|  | | ITO\|PEDOT:PSS\|**Com**:PVCz-PBD\|Cs:Al | | | 516, 552  516, 552 | | 519, 556  517, 555 | | | 4.5  6.5 | | 55 | |  |
| 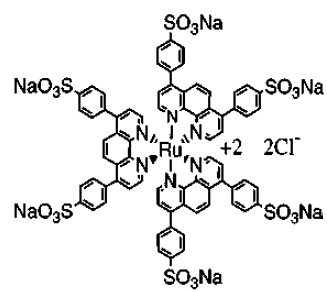 | | ITO/complex/Al | | | 628 | | orange-red light | | | 2.5 | | 56 | |  |
| 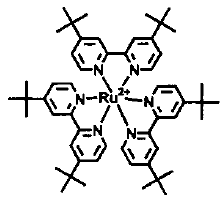 | | ITO/complex:PMMA/Ag | | | 630 | | ≈ 630 | | | 2.6 | | 57 | |  |
| 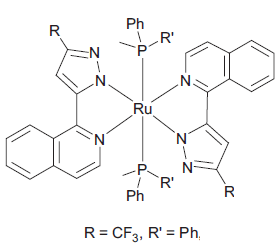 | | ITO/NPB/CBP:6% Ru complexes/BCP/AlQ3/  Mg:Ag. | | | 636 | | 626 | | | 3.7 | | 58 | |  |
| 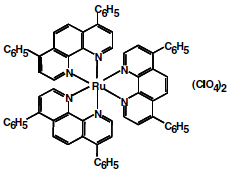 | | ITO/PVK:PBD:[Ru(dphphen)_3_]^+2^  (7%) 2þ/Alq3/Al | | | - | | ≈ 630 | | | 10-11 | | 59 | |  |
| 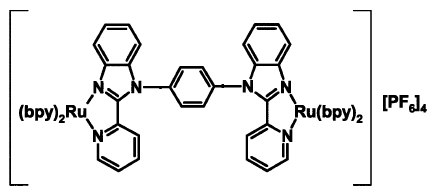 | | ITO/complex/Al | | | 630 | | 637 | | | 3.2 | | 60 | |  |
| 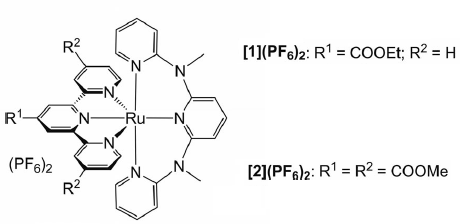 | | ITO/PEDOT:PSS/complex: /Ag | | | 729 | | 731 | | | 3 | | 61 | |  |
|  | |  | | | 744 | | 755 | | | 4 | |  | |  |
| 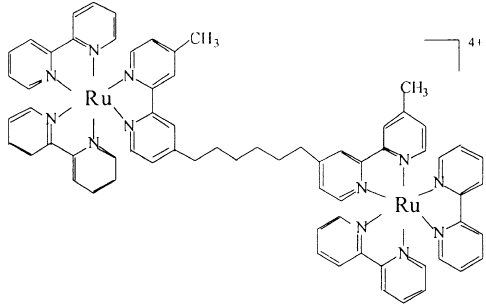 | | ITO/ Ru/ Li-triflate /Cr | | | - | | 638 | | | 6.8 | | 62 | |  |

**Table S2.** The energy levels of F1-F3 complexes calculated using LANL2DZ basis set in the solution phase (acetonitrile) based on the optimized S0 geometries.

| **Ruthenium complex** | **HOMO(eV)** | **LUMO (eV)** | **Band gap (eV)** |
| --- | --- | --- | --- |
| F1 | 6.06 | 2.79 | 3.26 |
| F2 | 5.98 | 2.73 | 3.23 |
| F3 | 6.05 | 2.75 | 3.28 |

**Table S3.** The main data of time resolved photoluminescence decay for F1-F3 complexes in ACN and film. The lifetime is obtained after using tri-exponential decay model through the following equation:

$$\tau_{Av}=\frac{\sum_{i} \tau_{i}A_{i}}{\sum_{i} A_{i}}$$

| **Complex No.** | **F1 in ACN** | **F1 as Film** | **F2 in ACN** | **F2 as Film** | **F3 in ACN (510 nm)** | **F3 as Film (526 nm)** | **F3 in ACN (625 nm)** | **F3 as Film (660 nm)** |
| --- | --- | --- | --- | --- | --- | --- | --- | --- |
| ***X*^2^** | 0.999 | 0.997 | 0.998 | 0.991 | 0.999 | 0.999 | 0.999 | 0.998 |
| **A_1_** | 0.07 | 0.599 | 0.12 | 0.764 | 0.20 | 0.579 | 0.43 | 0.914 |
| **τ_1_(ns)** | 0.0697 | 0.086 | 4.3882 | 0.241 | 0.0952 | 0.138 | 0.285 | 0.321 |
| **A_2_** | 0.08 | 0.338 | 0.26 | 0.209 | 0.55 | 0.405 | 0.28 | 0.0571 |
| **τ_2_(ns)** | 8.2033 | 0.491 | 41.5703 | 1.196 | 0.3783 | 0.357 | 2.7152 | 3.118 |
| **A_3_** | 0.85 | 0.0620 | 0.62 | 0.0261 | 0.25 | 0.0150 | 0.29 | 0.0286 |
| **τ_3_(ns)** | 59.9504 | 86.055 | 130.4097 | 200.756 | 2.7185 | 2.046 | 40.5086 | 160.136 |
| **τ_av_(ns)** | 51.619 | 5.5528 | 92.189 | 5.6738 | 0.907 | 0.53138 | 12.630 | 5.05132 |

**Table** S4.Comparison of lifetime of some polypyridyl Ruthenium complexes in solution and solid phase

| Complexes | sample | τ (ns) | Ref. |
| --- | --- | --- | --- |
| 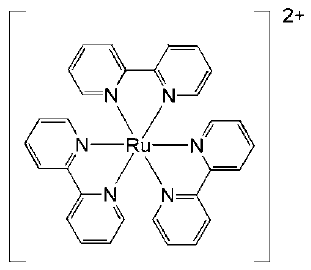 | Acetonitrile solution | 1100 | 11 |
|  | water | 358 | 12 |
|  | Coated on glass (dry) | 358 | 12 |
| 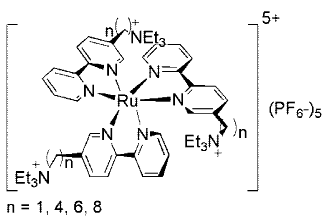 | Acetonitrile solution | n=1, 890 ns  n=4, 840 ns  n=6, 710 ns  n=8, 780 ns | 11 |
| 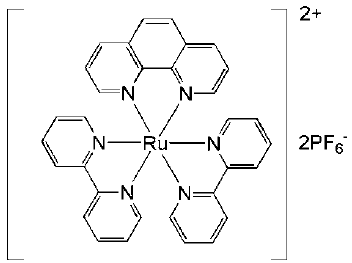 | Acetonitrile solution | 450 | 13 |
| 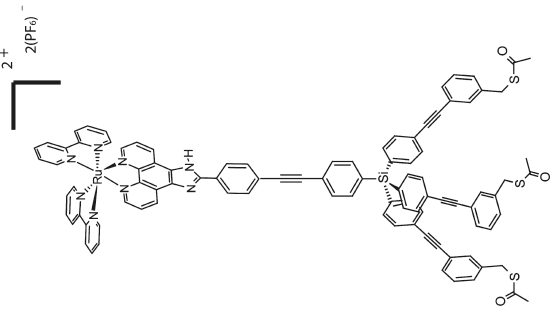 | Acetonitrile solution | 1040 | 14 |
|  | Coated on glass (dry) | 900 |  |
| 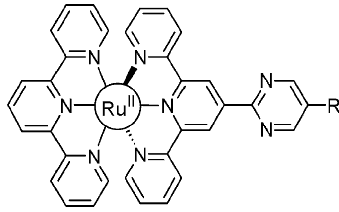 | Acetonitrile solution | R= H, 8 ns  R= CN, 200 ns  (λ_max_=675, 713) | 15 |
| 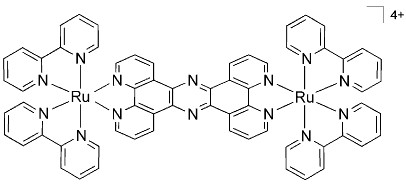 | Cell live | 175 | 16 |

**References**

# [1] M. Thelakkat and H. Schmidt, Adv. Mater., 1998, 10, 219.

# [2] R. S. Shraf, M. Shahid, E. Klemm and M. Al-Ibrahim, Macrol. Rapid. Commun. 2006, 27, 1454.

# [3] C. D. Sunesh, G. Mathai, and Y. Choe,Org. Elec., 2014, 15, 667.

# [4] M. M. Mandoc, G. A. KosterLP. W. M. Blom, Appl. Phys. Lett. 2007, 90, 133504.

[5] K. Suzuki,A. Kobayashi, S. Kaneko, K. Takehira, T. Yoshihara, H. Ishida, K. Shiina, S. Oishi, S. Tobita, Phys. Chem. Chem. Phys., 2009, 11, 9850.

[6] (a) S. Fantacci, F. De Angelis, A.Selloni, J. Am. Chem. Soc. 2003, 125, 4381.(b) S. Fantacci, F. De Angelis, A.Sgamellotti, Re, N. Chem. Phys.Lett. 2004, 396, 43.

[7] Sullivan, P. B.;Salmon, D. J.;Meyer, T. Mixed phosphine 2,2'-bipyridine complexes of ruthenium. Inorg. Chem. 1978, 17, 3334.

[8] J. P. Collin andJ. P. Sauvage, Inorg. Chem., 1986, 25, 135.

[9] R. Caspar, C. Cordier, J. B. Waern,C. G. Duhayon, M. Gruselle, P. Flochm and H. Amouri, Inorg. Chem. 2006, 45, 4071.

[10] Paq, W.; Eisenberg, R. Synthesis, characterization, and spectroscopy of dipyridocatecholate complexes of platinum. Inorg. Chem. **1997**,36, 2287.

[11] E.Zysman-Colman, J. D.Slinker, J. B.Parker, G.G.Malliaras, S.Bernhard, Chem. Mater. 2008, **20**, 388.

[12] E. L.Sciuto, M. F.Santangelob, G.Villaggio, S.F.inatra, C.Bongiorno, G.Nicotra, S.Libertinob, Sensing and Bio-Sensing Research, 2015, **6**, 67

[13] Sh.haomin Jis, W.Wu, W.Wu, P. Song, K. Han, Zh. Wang, Sh. Liu, H. Guo, J. Zhao, J. Mater. Chem., 2010, **20**, 1953

[14] S.Ramachandra, K. C.Schuermann, F.Edafe, P.Belser, C. A.Nijhuis, W. F.Reus, G. M.Whitesides, L. D.Cola, Inorg. Chem. 2011, **50**, 1581

[15] E. A.Medlycott, G. S.Hanan, Chem. Soc. Rev., 2005, 34, 133.

[16] E. Baggaley, M. R. Gill, N. H. Green, D. Turton, I. V. Sazanovich, S. W. Botchway, C. Smythe, J. W. Haycock, J. A. Weinstein, J. A.ThomasAngew. Chem. Int. Ed. 2014, 53, 3367.

[17] F. Dumur, D. Bertin, C. R. Mayer, A. Guerlin, G. Wantz, G. Nasr, E. Dumas, F. Miomandre, G. Clavier and D. Gigmes, *Synth. Met.*, 2011, **161**, 1934

[18] M. K. Nazeeruddin, R. Wegh, Z. Zhou, C. Klein, Q. Wang, F. De Angelis, S. Fantacci and M. Grätzel, *Inorg. Chem.*, 2006, **45**, 9245

[19] F. Dumur, G. Nasr, G. Wantz, C. R. Mayer, E. Dumas, A. Guerlin, F. Miomandre, G. Clavier, D. Bertin and D. Gigmes, *Org. Electron.*, 2011, **12**, 1683.

[20] A. Beeby, S. Bettington, I. D. Samuel and Z. Wang, *J. Mater. Chem.*, 2003, **13**, 80

[21]X. Wang, K. Ogino, K. Tanaka and H. Usui, *Selected Topics in Quantum Electronics, IEEE Journal of*, 2004, **10**, 121

[22] V. K. Rai, M. Nishiura, M. Takimoto, S. Zhao, Y. Liu and Z. Hou, *Inorg. Chem.*, 2011, **51**, 822

[23] F. Dumur, Y. Yuskevitch, G. Wantz, C. R. Mayer, D. Bertin and D. Gigmes, *Synth. Met.*, 2013, **177**, 100

[24]K. Chao, K. Shao, T. Peng, D. Zhu, Y. Wang, Y. Liu, Z. Su and M. R. Bryce, *J. Mater. Chem. C*, 2013, **1**, 6800

[25]X. Huixia, S. Peng, Z. Dan, Y. Tingting, H. Yuying, W. Hua, S. Heping and X. Bingshe, *New J. Chem.*, 2015.

[26] W.-Y. Wong, G.-J. Zhou, X.-M. Yu, H.-S. Kwok and Z. Lin, *Adv. Funct. Mater.*, 2007, **17**, 315.

[27] T. H. Kwon, Y. H. Oh, I. S. Shin and J. I. Hong, *Adv. Funct. Mater.*, 2009, **19**, 711

[28]H. Tsujimoto, S. Yagi, H. Asuka, Y. Inui, S. Ikawa, T. Maeda, H. Nakazumi and Y. Sakurai, *J. Organomet. Chem.*, 2010, **695**, 1972

[29]K. H. Lee, S. O. Kim, J. H. Seo, Y. K. Kim and S. S. Yoon, *J. Nanosci. Nanotechnol.*, 2011, **11**, 4471

[30]K. H. Lee, H. J. Kang, S. J. Lee, J. H. Seo, Y. K. Kim and S. S. Yoon, *Synth. Met.*, 2011, **161**, 1113

[31]D. Wang, J. Wang, H.-L. Fan, H.-F. Huang, Z.-Z. Chu, X.-C. Gao and D.-C. Zou, *Inorg. Chim. Acta*, 2011, **370**, 340

[32]S. Cao, L. Hao, W.-Y. Lai, H. Zhang, Z. Yu, X. Zhang, X. Liu and W. Huang, *J. Mater. Chem. C*, 2016, **4**, 4709

[33] K. H. Lee, H. J. Kang, S. J. Lee, Y. K. Kim and S. S. Yoon, *Synth. Met.*, 2012, **162**, 715

[34]C. L. Ho, C. S. Lam, N. Sun, D. Ma, L. Liu, Z. Q. Yu, L. Xue, Z. Lin, H. Li and Y. H. Lo, *Isr. J. Chem.*, 2014, **54**, 999

[35] S.-J. Yun, H.-J. Seo, M. Song, S.-H. Jin, S. K. Kang and Y.-I. Kim, *J. Organomet. Chem.*, 2013, **724**, 244

[36]Y. C. Zhu, L. Zhou, H. Y. Li, Q. L. Xu, M. Y. Teng, Y. X. Zheng, J. L. Zuo, H. J. Zhang and X. Z. You, *Adv. Mater.*, 2011, **23**, 4041

[37]Q.-L. Xu, C.-C. Wang, T.-Y. Li, M.-Y. Teng, S. Zhang, Y.-M. Jing, X. Yang, W.-N. Li, C. Lin and Y.-X. Zheng, *Inorg. Chem.*, 2013, **52**, 4916

[38]C. C. Wang, Y. M. Jing, T. Y. Li, Q. L. Xu, S. Zhang, W. N. Li, Y. X. Zheng, J. L. Zuo, X. Z. You and X. Q. Wang, *Eur. J. Inorg. Chem.*, 2013, **2013**, 5683

[39]M.-Y. Teng, S. Zhang, Y.-M. Jin, T.-Y. Li, X. Liu, Q.-L. Xu, C. Lin, Y.-X. Zheng, L. Wang and J.-L. Zuo, *Dyes Pigm.*, 2014, **105**, 105

[40]S.-J. Yun, H.-J. Seo, M. Song, S.-H. Jin and Y. I. Kim, *Bull. Korean Chem. Soc*, 2012, **33**, 3645.

[41] L. He, J. Qiao, L. Duan, G. Dong, D. Zhang, L. Wang and Y. Qiu, *Adv. Funct. Mater.*, 2009, **19**, 2950

[42]F. Zhang, L. Duan, J. Qiao, G. Dong, L. Wang and Y. Qiu, *Org. Electron.*, 2012, **13**, 1277-

[43] B. Umamahesh, N. Karthikeyan, K. Sathiyanarayanan, J. Malicka and M. Cocchi, *J. Mater. Chem. C*, 2016, **4**, 10053

[44] J. S. M. Fernández-Hernández, J. I. Beltrán, V. Lemaur, M.-D. Gálvez-López, C.-H. Chien, F. Polo, E. Orselli, R. Fröhlich, J. r. m. Cornil and L. De Cola, *Inorg. Chem.*, 2013, **52**, 1812

[45]H.-Y. Li, T.-Y. Li, M.-Y. Teng, Q.-L. Xu, S. Zhang, Y.-M. Jin, X. Liu, Y.-X. Zheng and J.-L. Zuo, *J. Mater. Chem. C*, 2014, **2**, 1116

[46]F. Zhang, D. Ma, L. Duan, J. Qiao, G. Dong, L. Wang and Y. Qiu, *Inorg. Chem.*, 2014, **53**, 6596

[47]N. M. Shavaleev, G. Xie, S. Varghese, D. B. Cordes, A. M. Slawin, C. Momblona, E. Ortí, H. J. Bolink, I. D. Samuel and E. Zysman-Colman, *Inorg. Chem.*, 2015, **54**, 5907

[48] P. T. Furuta, L. Deng, S. Garon, M. E. Thompson and J. M. Fréchet, *J. Am. Chem. Soc.*, 2004, **126**, 15388

[49] A. S. Ionkin, W. J. Marshall and Y. Wang, *Organometallics*, 2005, **24**, 619.

[50]W.-Y. Wong, Z. He, S.-K. So, K.-L. Tong and Z. Lin, *Organometallics*, 2005, **24**, 4079.

[51]Z. He, W.-Y. Wong, X. Yu, H.-S. Kwok and Z. Lin, *Inorg. Chem.*, 2006, **45**, 10922.

[52]G.-J. Zhou, W.-Y. Wong, B. Yao, Z. Xie and L. Wang, *J. Mater. Chem.*, 2008, **18**, 1799.

[53]C.-L. Ho, W.-Y. Wong, B. Yao, Z. Xie, L. Wang and Z. Lin, *J. Organomet. Chem.*, 2009, **694**, 2735.

[54]Y. E. Begantsova, L. N. Bochkarev, I. P. Malysheva, N. E. Stolyarova, Y. A. Kurskii, M. A. Lopatin, E. V. Baranov, V. A. Ilichev, G. A. Abakumov and M. N. Bochkarev, *Synth. Met.*, 2011, **161**, 1043.

[55]T. Shigehiro, S. Yagi, T. Maeda, H. Nakazumi, H. Fujiwara and Y. Sakurai, *J. Phys. Chem. C*, 2012, **117**, 532.

[56] J.-K. Lee, D. S. Yoo, E. S. Handy & M. F. Rubner, Appl. Phys. Lett., Vol. 69, No. 12 (1996).

[57] Hartmut Rudmann, Satoru Shimada, and Michael F. Rubner, J. AM. CHEM. SOC. 2002, 124, 4918.

[58]Yung-Liang Tung, Li-Shiuan Chen, Yun Chi, Pi-Tai Chou, Yi-Ming Cheng, Elise Y. Li, Gene-Hsiang Lee, Ching-Fong Shu, Fang-Iy Wu, and Arthur J. Carty, Adv. Funct. Mater. 2006, 16, 1615.

[59]Jihua Yang, Keith C. Gordon, Chemical Physics Letters 385, 2004, 481.

[60]Wen-Li Jia, Yu-Feng Hu, Jun Gao and Suning Wang, Dalton Trans., 2006, 1721

[61] Aaron Breivogel, Myeongjin Park, Donggu Lee, Stefanie Klassen, Angelika Kühnle, Changhee Lee, Kookheon Char, and Katja Heinze, Eur. J. Inorg. Chem. 2014, 288

[62]Lepretrea, J.-C., Deronziera, A. & Stephan, O., Synth. Met.,2002, **131**, 175
